# Remote sensing and computer vision for marine aquaculture

**DOI:** 10.1126/sciadv.adn4944

**Published:** 2024-10-16

**Authors:** Sebastian Quaade, Andrea Vallebueno, Olivia D. N. Alcabes, Kit T. Rodolfa, Daniel E. Ho

**Affiliations:** ^1^ Regulation, Evaluation and Governance Lab, Stanford University, Stanford, CA, USA.; ^2^ California Institute of Technology, Pasadena, CA, USA.

## Abstract

Aquaculture, the cultivation of aquatic plants and animals, has grown rapidly since the 1990s, but sparse, self-reported, and aggregated production data limit the effective understanding and monitoring of the industry’s trends and potential risks. Building on a manual survey of aquaculture production from remote sensing imagery, we train a computer vision model to identify marine aquaculture cages from aerial and satellite imagery and generate a spatially explicit dataset of finfish production locations in the French Mediterranean from 2000 to 2021 including 4010 cages (average cage area, 69 square meters). We demonstrate the value of our method as an easily adaptable, cost-effective approach that can improve the speed and reliability of aquaculture surveys and enables downstream analyses relevant to researchers and regulators. We illustrate its use to compute independent estimates of production and develop a flexible framework to quantify uncertainty in these estimates. Overall, our study presents an efficient, scalable, and adaptable method for monitoring aquaculture production from remote sensing imagery.

## Teaser

Object detection methods on remote sensing imagery can be effective tools to monitor aquaculture production around the world.

## INTRODUCTION

Aquaculture, the cultivation of aquatic plants and animals, has recently emerged as a key driver of the supply of aquatic foods. With a sixfold increase in global production between 1990 and 2020 (*1*), it is the fastest growing food production sector globally (*2*, *3*), outputting 122.6 million tonnes (live weight) in 2020 across freshwater, brackish water, and marine culture systems (*1*). This rapid development and the strengthening of its role as a key contributor to seafood supply have underscored the debate around aquaculture’s social and environmental impact. On the one hand, the industry has been touted for its potential to increase food security and nutrition (*4*–*7*). In 2018, aquatic animals provided 15.3% of global crude protein supply, with 7.4% stemming from aquaculture production (*8*), and estimates indicate that aquatic food production could contribute up to 8% of zinc and iron and 27% of vitamin B12 global supply in 2030 (*6*). At the same time, aquaculture is associated with a number of environmental harms. Animal waste and inefficient feeding can cause algal bloom outbreaks that adversely affect water quality for humans and broader ecosystem health (*9*–*12*). In addition, the industry’s use of antimicrobials to manage disease outbreaks, which is estimated to exceed human and terrestrial animal consumption levels (*13*), has been found to contribute to the emergence of multi-antimicrobial resistant strains (*14*). Moreover, several scholars have raised concerns regarding the welfare of intensively farmed aquatic animals (*15*–*18*).

Aquaculture’s rapid growth and environmental footprint has increased demand for timely and reliable data on the industry. The Food and Agriculture Organization (FAO) generates the only source of data on fisheries and aquaculture production at a global level (*1*). On an annual basis, it publishes country-level aquaculture production data using data primarily assembled from surveys conducted by national statistical offices (*19*). While FAO leverages alternative data sources to compile these monitoring statistics as accurately as possible, their reliance on national reports, which can depend on producers to self-report production amounts (*20*), implies that the quality and availability of data are highly related to that of the surveys. In addition to general non-reporting, these can suffer from partial information, inconsistencies, highly aggregated data that fail to meet reporting standards, and varying data quality across species and production systems (*1*). Leading aquaculture nations have in the past been found to misreport production statistics (*21*). In 2020, FAO indicated that only 59% of countries submitted or reported official aquaculture data, although these represented 97.6% of global production (*1*).

Spatially granular and disaggregated data on aquaculture production is key to effective governance, monitoring, and regulation. Many of aquaculture’s negative environmental effects, including nutrification, chemical pollution, and threats to marine mammals (*22*–*24*), can be highly localized, and its environmental impact varies across production systems (*25*). Production primarily includes (i) inland aquaculture (44% of global production in 2020), which takes place in freshwater systems located in waterways such as rivers, ponds, or canals; and (ii) marine aquaculture (“mariculture,” 47% of production), which cultivates species at sea during the entirety of the life cycle or exclusively during the grow-out stage (*1*, *26*). However, few sources of data specify the location and intensity of mariculture with high spatial resolution. The European Marine Observation and Data Network compiles data on marine finfish production locations using government reports from European countries (*27*). However, only Cyprus, Denmark, Finland, Greece, Ireland, Malta, Norway, Spain, and Scotland report such data. More ambitiously, Clawson and colleagues (*28*) derive a spatially detailed dataset of global mariculture production by aggregating aquaculture data from national data releases, peer-reviewed research, and data releases from industry and research organizations. Although they are able to allocate 96% of mariculture production to specific spatial locations, only 17% of farms in their dataset have known locations, while the locations of the remaining 83% are imputed. Furthermore, at a resolution of 25 km^2^, the dataset may not be sufficiently granular to assess the effects of mariculture effluents that are spatially concentrated. To derive higher-resolution distributions of mariculture production across space, more data are needed on the location and size of these farms.

One way to obtain precise marine aquaculture locations is to identify farms from remote sensing imagery. For instance, Trujillo *et al*. (*29*) and Katselis *et al*. (*30*) manually scan the Mediterranean and Greek coasts, respectively, using Google Earth to develop bottom-up inventories of marine aquaculture production locations. Manual labeling efforts like these can provide valuable detailed information. At the same time, they are highly time-consuming and do not easily replicate or scale over time and space. A large literature uses a variety of classical spectral, spatial features analysis, and object-based image analysis methods to classify aquaculture production areas in satellite imagery [see for instance, (*31*) and (*32*)]. However, many of these methods rely on a small number of tunable parameters, meaning that their performance can be negatively affected when aquaculture facilities and ocean backgrounds vary in appearance (*33*).

More recent work takes advantage of developments in computer vision to improve the accuracy of automated methods for mapping food production from remote sensing imagery. In the United States, deep learning approaches, namely, object detection and segmentation models, have been used to locate land-based agriculture facilities that have not been previously reported (*34*, *35*). Several studies have also used deep learning to map mariculture in China. For example, a variety of image segmentation models have been trained to recognize aquaculture areas in low to medium resolution images, ranging from specific regional maps to mapping all mariculture facilities along the Chinese coast (*36*–*39*). Other researchers have trained image segmentation models to map mariculture farms on high-resolution imagery, although they have resorted to small study areas due to limited data availability (*33*, *40*). These studies have been primarily methodological in nature, designing their own deep learning architectures to enhance models’ capacity to segment aquaculture rafts. The literature has underexplored whether off-the-shelf deep learning methods—such as widely available, pretrained object detection models—can be readily deployed by researchers to monitor aquaculture production with reasonable performance. Moreover, to our knowledge, deep learning–based approaches for identifying marine finfish production have not been developed for other regions around the world.

**Fig. 1.**
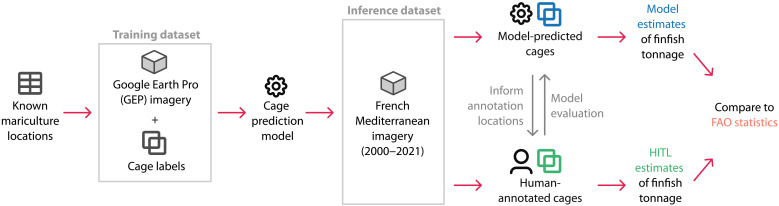
Overview of our approach. First, we train a model to predict finfish cages from remote sensing imagery, using a dataset of GEP imagery and cage bounding boxes around known mariculture locations. Then, we obtain cage polygons on 2000–2021 aerial imagery of the French Mediterranean provided by IGN from two sources: first, from our model; second, from human annotations of a model-informed subset of the imagery, which are also used to evaluate our model’s performance. Last, we compute estimates of finfish mariculture production in the region using both the predicted cages (model estimates) and the human-annotated cages [human-in-the-loop (HITL) estimates] and compare these to FAO statistics.

To address the scarcity of high-resolution geospatial mariculture data, we develop a computer vision–based method for identifying marine finfish farms from remote sensing imagery. Building on a manual mapping of ocean fish farms by Trujillo *et al.* (*29*), we first create a hand-labeled dataset of mariculture finfish cages on high-resolution satellite and aerial imagery from Google Earth Pro (GEP) (*41*). Next, we train a model to predict individual cages in remote sensing images by fine-tuning a YOLOv5 object detection model on this dataset and applying a cage detection post-processing procedure. We then evaluate our model’s performance and demonstrate the value of our method for aquaculture monitoring in a representative setting, the entire French Mediterranean coast, using publicly available aerial imagery from Institut national de l’information géographique et forestière (IGN) (*42*). To illustrate the impactful downstream analyses that can be performed with our method, we create an inventory of marine finfish facilities and facility size in this region over time. Moreover, we generate production estimates for the region, with accompanying uncertainty measures, and compare these temporal estimates to survey-based data from FAO. Figure 1 presents an overview of our approach. Overall, our work (i) presents a cost-effective and adaptable methodology for offshore aquaculture detection; (ii) demonstrates a procedure for estimating marine finfish production from remote sensing imagery, with appropriate measures for uncertainty from object detection; and (iii) showcases the use of our method as part of a human–artificial intelligence collaborative system that experts can use to conduct more efficient aquaculture surveys.

## RESULTS

### A computer vision model to detect aquaculture cages

**Fig. 2.**
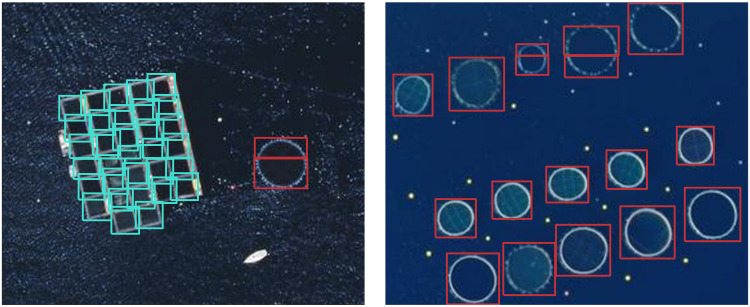
Example detections of aquaculture cages. Detections of surface square (cyan) and circular (red) finfish aquaculture cages identified by our object detection model on aerial imagery of the French Mediterranean. Imagery: IGN (*42*).

Offshore finfish aquaculture primarily uses floating cage structures, which are often placed in clusters near feed storage, operation platforms, and other central resources. As the cage structures belong to two main typologies (square or circular) with differing geometries, we opted for a computer vision model that could not only detect the cage instances but also identify their typology. This enables downstream calculations of the cage area using the detected bounding boxes and, thus, the estimation of production tonnage. Figure 2 illustrates these different types of cage structures and showcases our model’s ability to detect and distinguish the two typologies.

**Fig. 3.**
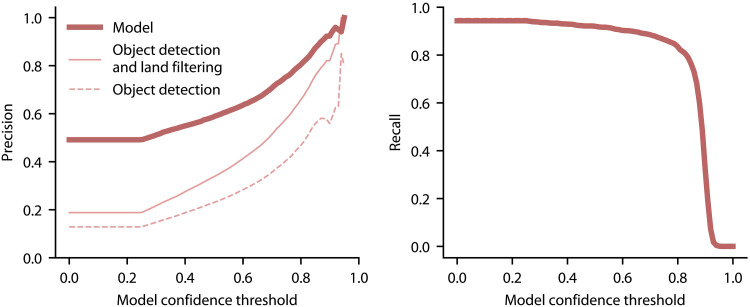
Performance (cage-level precision and recall) on the French Mediterranean coast. The dark line reflects performance of the overall methodology (i.e., the object detection model in addition to the removal of land-based detections and cage clustering), whereas lighter lines reflect the stand-alone performance of the object detection model without these sequential postprocessing steps. Recall is virtually unchanged across the detection model and post-processing steps.

We evaluate our method’s performance along the entire French Mediterranean coast, a representative setting of a region of interest for aquaculture activity detection, on aerial imagery from 2000 to 2021 using the sampling approach described in the Materials and Methods (see the “Inference data collection” and “Model performance evaluation” sections). Our overall methodology involves obtaining predictions from an object detection model, followed by two post-processing steps. The first step removes land-based objects by using a geometry of the French coastline to filter out all detections that are not located in the ocean. The second step clusters the cage-level predictions into cage groups similar to the structures that are typically operated by aquaculture facilities. As we are unable to determine whether cage groups fall under the same ownership solely from the aerial imagery, we refer to these sets of cages as clusters rather than facilities. This clustering is based on cage proximity and the number of cages within the cage cluster and is an effective approach to remove isolated predictions, which are often false positive. Figure 3 visualizes our method’s performance across model confidence scores on the complete coastal imagery of the region. At a confidence threshold of 0.80, it achieves cage-level precision and recall of 82%. Moreover, this figure illustrates the considerable value of the methodology’s post-processing steps for overall precision, particularly when compared to the stand-alone object detection model (see the Supplementary Materials for typical false-positive predictions; fig. S1). We tuned this post-processing procedure via a cross-validation approach using the hyperparameter grid search procedure described in the Supplementary Materials. The tuned prediction model achieves 91% precision and 73% recall at the cage level and 82% precision and 79% recall at the cage cluster level, on an independent test set comprising 10% of the region’s aerial imagery of the ocean.

**Fig. 4.**
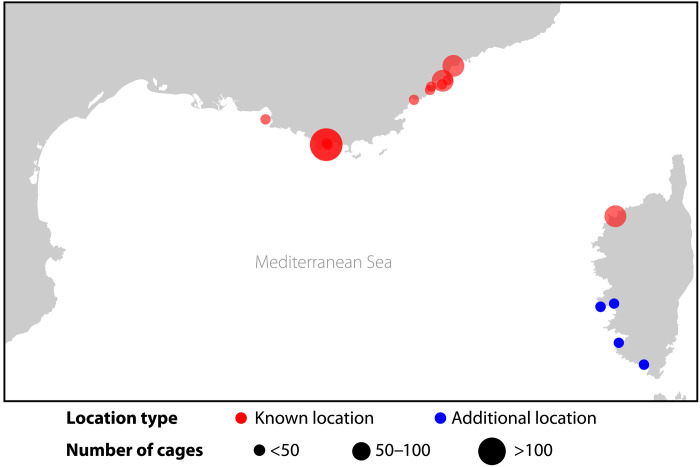
Marine finfish aquaculture production locations in the French Mediterranean. Red points indicate the known locations found by Trujillo *et al.* (*29*) in their manual survey of Google Earth during 2002–2010. Blue points indicate cage clusters detected by our model during 2000–2021 that are at least 1 km away from these known locations.

Our methodology’s ability to effectively locate aquaculture production is underlined by its performance relative to manual labeling efforts. Because of the cadence of the French aerial imagery (see the “Inference data collection” section), there is a slight mismatch between the study period of Trujillo *et al.*’s (*29*) manual survey of the region (2002–2010) and the timing of our imagery. Nonetheless, we make the closest comparison possible by considering cage clusters from 2000 to 2004, 2005 to 2009, and 2010 to 2012 to find new locations within Trujillo *et al.*’s (*29*) time period and cage clusters from 2013 to 2015, 2016 to 2018, and 2019 to 2021 to find new locations that postdate their study period. In this manner, we find all of the marine finfish aquaculture locations in the French Mediterranean that were identified by Trujillo *et al.* (*29*) in their manual survey of Google Earth from 2002 to 2010. During this study period, our model also finds an additional seven cage clusters that are more than 1 km away from Trujillo *et al.*’s (*29*) identified locations. Furthermore, we identify an additional eight clusters that postdate their study period (on imagery from 2013 to 2021) and are more than 1 km away from their identified locations. Figure 4 maps the known marine finfish aquaculture locations identified by Trujillo *et al.* (*29*) and the cage clusters detected by our model during 2000–2021 that are located at least 1 km away from these known production locations (see the Supplementary Materials for a visualization of these locations over time; fig. S2).

### Aquaculture production estimation in the French Mediterranean

**Fig. 5.**
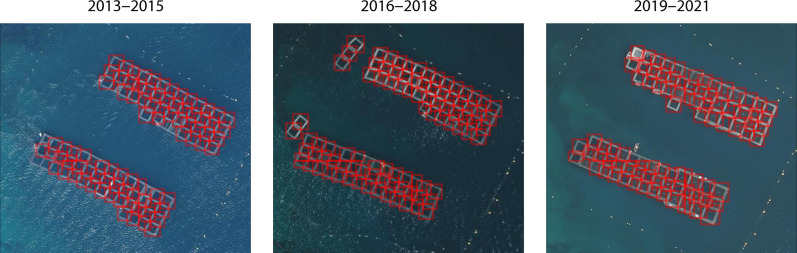
Predictions for a cluster of cages in the French Mediterranean over time. Imagery: IGN (*42*).

Our method allows us to directly estimate the number of aquaculture farms at a given point in time, as well as the number and surface area of cages at each location (Fig. 5). One downstream analysis these predictions can be particularly useful for is developing a bottom-up estimate of finish production that is almost independent of self-reported and potentially incomplete survey-based estimates. To illustrate this application, we compute annualized estimates of finfish mariculture production in the French Mediterranean for the 2000–2021 period using our method and compare these estimates to FAO statistics reported for the same species, region of interest and time period. Because of the cadence of the French aerial imagery, we compute production estimates over time periods corresponding to imagery waves over the French coast and estimate the annualized production over each wave of imagery on the assumption that production is relatively stable over this time frame.

**Fig. 6.**
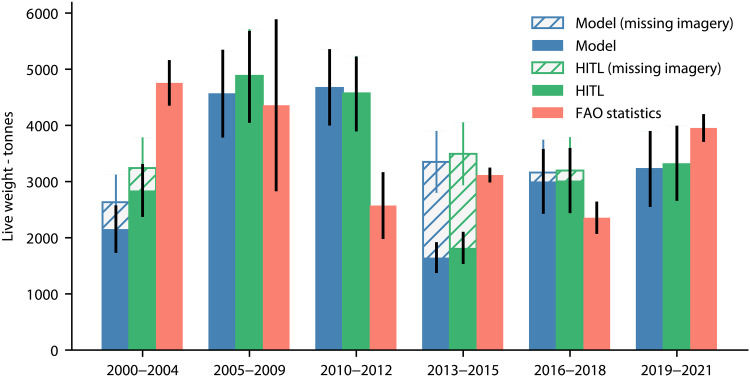
Marine finfish aquaculture tonnage in the French Mediterranean over time. Model estimates reflect annualized finfish tonnage computed from the area of model-predicted cages, with error bars reflecting SD measures that incorporate uncertainty in the aerial imagery, in model performance, in cage area estimates, and in the tonnage production factors. HITL estimates reflect annualized finfish tonnage computed from the area of human-annotated cages, with error bars reflecting SD measures that incorporate uncertainty in the aerial imagery, in cage area estimates, and in the tonnage production factors. FAO data reflect average annual finfish production reported to FAO during the period, with error bars reflecting the SD of these values. We include imputed estimates of annualized finfish production that account for missing aerial imagery in some locations (see the “Production estimation and uncertainty measurement” section).

Figure 6 visualizes the annualized finfish production values (measured in tonnes, live weight equivalent) estimated by our method (blue) and the average annual finfish production values reported by FAO (orange) for each time period. In the case of our model estimates, the error bars reflect SD measures that account for the following sources of uncertainty: (i) model performance; (ii) differing cage area estimates arising from multiple aerial images for a given location; (iii) cage area uncertainty from detected bounding boxes; and (iv) uncertainty in other factors of production (cage depth, stocking density, and harvest frequency), modeled according to the distributions described in the Supplementary Materials (fig. S3). For FAO statistics, error bars reflect the SD of the annual production statistics that fall in each time period. We also include imputed tonnage estimates, reflecting average production over a broader time frame, that account for the fact that the aerial imagery is unavailable in some locations (see the “Production estimation and uncertainty measurement” section for more details on the production estimation and imputation methodologies).

Overall, we find that our tonnage estimates match up quite closely to FAO statistics in most periods, with two exceptions. In 2000–2004, our estimates are 55% lower, driven by lower quality of the coastal imagery that is available for these years, limiting the ability of our model to identify cages. On the other hand, our estimates are 82% higher in 2010–2012. The stark decline in French finfish tonnage exhibited during 2005–2009, likely driven by weakened demand at the time of the economic crisis (*43*) and reflected in Fig. 6 in the wide error bars for the FAO data in this period, could be an explanation for this discrepancy. It is possible that retired cages are not immediately removed from the water, such that our estimates for the period include the estimated tonnage of a large number of cages that became inactive during this time of rapid production decline. In addition to FAO statistics, we can compare our tonnage estimates to those of Trujillo *et al*. (*29*), who also use the cage areas, derived from their manual survey, to estimate production. Within 1 km of the authors’ known facilities, we estimate annualized finfish production of 2590 (SD of 549) tonnes during the 2005–2009 period, closely tracking their estimate of 2678 tonnes (assuming that 100% of cages are in production) produced in 2006.

While our model can provide a rapid and robust means of identifying aquaculture locations and measuring their extent and production, one option for improving further on the accuracy of these measurements is to use our method as a means of identifying candidate locations for human review. Such a human-in-the-loop (HITL) approach emphasizes the use of human interaction to more effectively test models and to produce more relevant model outputs. In our application, this framework takes advantage of the domain expertise and judgement that benefits manual surveys of remote sensing imagery while markedly reducing the burden of these efforts. By focusing human annotation efforts on images that have a higher likelihood of containing cages (as indicated by the model), human reviewers can perform a thorough survey of a region while looking at only a fraction of the area’s imagery. For instance, in our French Mediterranean setting, human reviewers only looked at 3.6% of the entirety of the coastal imagery to confirm all of the model predictions and to estimate an upper bound on the total number of cages in the region. In this manner, this framework represents a highly cost-effective and accurate alternative to existing manual efforts.

Figure 6 also visualizes tonnage estimates (green) computed from the set of human-annotated cages that was derived from examining only the images in which the model detected a cage (with any confidence score), images near known aquaculture production locations, and a small sample of images that neither have predictions nor are close to known locations. While the HITL estimates are similar to the model-derived estimates in general, reflecting the overall high quality of the model’s predictions, HITL estimates are notably higher in the 2000–2004 period where poor image quality limited the model’s performance. Nonetheless, these estimates remain considerably below the average FAO-reported tonnage for that period, suggesting that the image quality issues may still pose a challenge for the human reviewers as well as the model. As a separate exercise, we can also use the HITL approach to estimate an upper bound on the total number of cages in the coastal imagery from 2000 to 2021. We estimate a population of 4285 cages, including 4010 cages that were manually annotated from 3.6% of the imagery, and 275 cages estimated from the remaining, unannotated portion of the imagery.

## DISCUSSION

We have developed a highly adaptable and cost-effective framework that leverages high-resolution remote sensing imagery and object detection to locate finfish mariculture locations and estimate their production over time with robust uncertainty measures. We have demonstrated the application of our approach in the context of the French Mediterranean, representing, to our knowledge, the first deep learning–based mariculture mapping application outside of China. Our method exhibits strong performance on our independent and representative test set from the French Mediterranean, with 91% precision and 73% recall at the cage level. It finds all of the known production sites in the region that have been identified in a prior manual survey by Trujillo *et al.* (*29*) and discovers additional locations. Moreover, we have found that our production estimates capture at minimum the mariculture finfish tonnage reflected in FAO statistics for the region in all but one time period, potentially due to poor aerial image quality in 2000–2004.

Our methodology has a number of potential applications for environmental monitoring and impact studies. First, it enables the acquisition of data on mariculture production sites in a region, as demonstrated along the French Mediterranean coast, as well as their locations and sizes. These data are highly relevant for understanding the spread and scale of aquaculture production at any given point in time and, when compared inter-temporally, can give insight into industry dynamics. Second, it offers an alternative to estimates of aquaculture production based on producer surveys, with appropriate measures of uncertainty that account for variance in the object detection step. Remotely sensed aquaculture data have the potential to allow experts to estimate production where survey data are missing and can be used to verify survey-based data where it is available. Notably, our method can be used to compute these estimates in a way that maintains a high level of expert involvement. We exemplified this approach with the manual annotation of fewer than 4% of the entire set of images that was viewed by our model to produce temporal estimates for the French Mediterranean coast during a 21-year period.

**Fig. 7.**
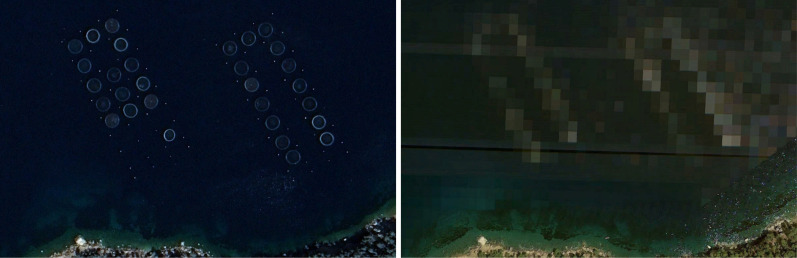
Pixelated imagery. Remotely sensed imagery for an aquaculture facility near the Greek island of Poros from April 2019 (left) and June 2021 (right). Imagery: Google, 2023 CNES/Airbus (April 2019 image) (*41*).

While our approach offers numerous advantages, we note several limitations to the current work. First, our approach may not generalize to other regions that use different structures for mariculture production. For instance, our method is unsuitable for detecting underwater mariculture rafts, which are difficult to identify from remote sensing imagery. While research interest in submersible cages has increased over the past couple of decades, our method’s relevance is underpinned by the fact that surface cage culture remains the dominant production typology, and not all finfish species are well suited to submersible culture (*44*). Second, our method is dependent on the availability of high-resolution aerial or satellite imagery, and limited image cadence may limit the granularity of any longitudinal study. In our estimation of mariculture production in the French Mediterranean, the available imagery limited our results to a cadence of 3 to 4 years over the 2000–2021 period, and lower quality of the imagery from earlier years affected the performance of our model. The limitations of relying on high-resolution imagery extend to other production systems that our method could potentially be adapted to monitor. For instance, the extraction of inland aquaculture ponds used in freshwater aquaculture [44% of global aquaculture production, of which 57% is in China; (*26*)] typically relies on high-resolution imagery given the narrow dikes that separate these ponds, difficulties distinguishing these from other water bodies, and the complex land cover contexts in which these are located (*45*–*47*). In the context of detecting mariculture cages, the resolution must be high enough to allow to clearly distinguish mariculture cages from other ocean features and, therefore, depends on the size and morphology of cages in a given region. The availability of high-resolution imagery of global coastlines and seas varies substantially across time and geography but continued improvements in the availability, cadence, and quality of satellite data (*48*) suggest that these kinds of tools are likely to become more useful over time. Separately, we noticed several instances of mariculture farms that were pixelated to the point of obfuscation in our images. Some of these instances, such as the imagery of an aquaculture facility near the Greek island of Poros (Fig. 7), have arisen after community-guided efforts have pointed to the adverse environmental effects of these farms (*49*). This kind of alteration to remote sensing imagery can affect the usefulness of imagery-based methods for environmental monitoring. Third, there are inherent limitations to measuring the magnitude of aquaculture tonnage with a high level of precision using this object detection on remote sensing imagery approach. For instance, our area computations are necessarily based on bounding box predictions for individual cages rather than a precise outline of the cage objects (see the “Production estimation and uncertainty measurement” section). Although we account for uncertainty stemming from the imprecision of bounding boxes in our estimates, cage predictions from image segmentation may result in greater precision. In addition, our method does not identify the extent to which cages are used during observation periods, which means that our estimates may better reflect available production capacity than actual production during periods of low utilization. Fourth, even with perfectly accurate cage predictions, computing production tonnage from these annotations is a challenging task due to the need for alternative data sources (e.g., bathymetry for cage depth, stocking densities, and harvest frequencies), an understanding of local regulation and enforcement, and the domain expertise required to model the production factor distributions to calculate sound uncertainty measures. Although our uncertainty estimates do not model more complex relationships such as the correlation of production factors across facilities, we note that our framework can be easily adapted to incorporate more complex mechanisms of this nature.

This study focuses on mariculture in the French Mediterranean as a proof of concept for a framework that can be adapted to other settings. With a small amount of training data capturing the dominant cage typologies used in this location and quick fine-tuning of a pretrained detection model, our approach enabled the comprehensive estimation of the region’s mariculture production. In the same manner, we expect that our approach can generalize and be readily implemented in new locations by collecting a modest amount of labels that are representative of the region’s cage farms and retraining the model with these data. Our aim is that the highly adaptive nature of our approach can lower barriers to using deep learning for the environmental monitoring of aquaculture and make these tools more accessible to researchers with less experience using deep learning methods. Our tonnage estimation procedure, which applied the method of composition to capture uncertainty in our estimates stemming from multiple sources, also demonstrates an uncertainty quantification approach that can be adapted to other aquaculture settings and even to machine learning–based work seeking to compute production estimates via remote sensing in general. To enable other researchers to build on our work, we release a dataset of aerial images of the French Mediterranean from IGN’s BD Ortho series (*42*) and a dataset of remotely sensed images of the Mediterranean from Google Earth (*41*), available to academic researchers upon request, that can be used to implement our method in other producing regions. In combination, these datasets contain ~6500 square and circular surface cage bounding box annotations.

To conclude, in this study, we developed a method to detect aquaculture production locations from remote sensing using an object detection approach, present the first instance (to our knowledge) of using deep learning to detect mariculture farms outside of China, and demonstrate how our method can be used to detect aquaculture sites in the French Mediterranean. Our aim is that this highly adaptable and accessible approach can markedly improve the capacity of aquaculture researchers and regulators to monitor industry dynamics and detect environmental harms, build public awareness around these issues, and build an evidence base for improvements in regulations that balance needs for food security, environmental impact, and animal welfare.

## MATERIALS AND METHODS

**Fig. 8.**
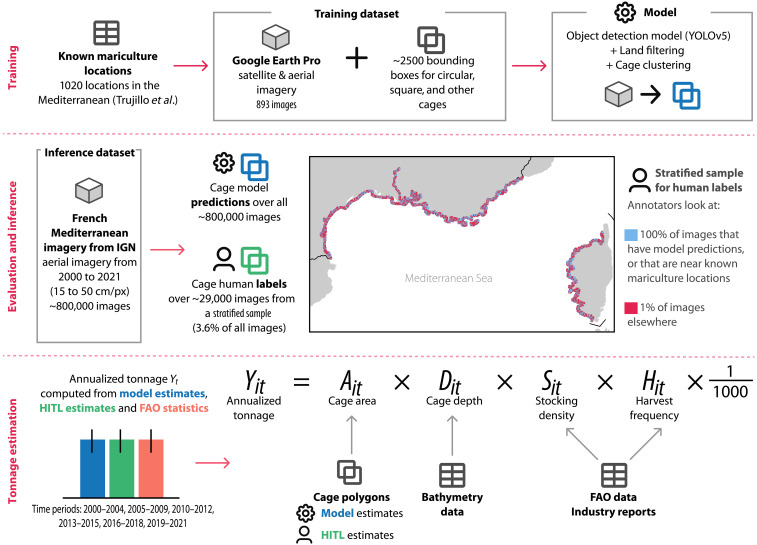
Methodology overview. Our approach consists of the following steps. First, we use known mariculture locations in the Mediterranean to create a training dataset of mariculture cages from GEP imagery and train a model on this dataset to predict cage bounding boxes from remote sensing imagery. Second, we run our model on aerial imagery of the French Mediterranean from 2000 to 2021. In addition, we ask a team of human labelers to annotate 3.6% of this French aerial imagery (per a stratified random sample) and use these human labels to evaluate model performance. Third, we compute two kinds of annualized tonnage estimates and uncertainty measures: (i) model estimates, using the predicted cages; and (ii) HITL estimates, using the human labels; and we compare both to FAO statistics.

The subsections below describe the key components of our marine aquaculture prediction approach: first, the data collection, model training, and the output post-processing procedures that we used to develop our prediction model that predicts cage bounding boxes from remote sensing imagery; and, second, the data collection, model evaluation, and statistical methods that we used to locate and estimate marine finfish aquaculture production in the French Mediterranean from aerial imagery using our prediction model. In the Supplementary Materials, we include additional details describing the cage annotation protocol used to build the training and evaluation datasets, the method used to evaluate model performance, the approach used to tune our prediction post-processing procedure, our method to estimate cage areas from bounding box predictions (see table S1), and the production factor distributions used in our tonnage uncertainty quantification methodology (see fig. S3). Figure 8 presents an overview of our methodology, as well as the imagery, external datasets, and cage inputs that we use at each step.

### Training a computer vision model to detect finfish cages from remote sensing imagery

#### Training data collection

To obtain training data for our computer vision model, we identified Mediterranean marine aquaculture sites, using a dataset of 1020 locations manually assembled by Trujillo *et al.* (*29*), in GEP imagery (*41*). The facilities in this dataset are primarily located in the coastal waters of Greece, Italy, Croatia, France, Albania, and Malta. GEP includes aerial, satellite, and other types of remotely sensed images that are collected from different providers. In the locations that we explored to extract high-resolution imagery for our training data, GEP images were primarily sourced from Maxar Technologies and Centre National D’Etudes Spatiales (CNES)/Airbus, a partnership between Airbus and the French Space Agency (CNES). There is no systematic approach to determine the resolution of the collected images; however, we found that the Maxar-sourced images for some locations were derived from the WorldView-2 (resolution of ~48 cm/pixel) and WorldView-3 (resolution of ~34 cm/pixel) satellites.

We used GEP’s historical imagery feature to download large, high-resolution images at each location for all available time snapshots. Specifically, at each location, we standardized the GEP view to be centered at the location’s coordinates with an altitude of 0 m, a tilt of 0°, and a range of 700 m and downloaded an image at each available time snapshot at the highest pixel resolution possible (8192 × 5673) given our hardware’s screen dimensions. The images covered an extent of 810 m by 558 m, resulting in a resolution of ~9 to 10 cm/pixel for the exported images.

To pre-process the raw images that we downloaded, we downscaled them by 50% and tiled them into 1024 × 1024 pixel images. Then, we randomly drew from these tiled images to hand-label cages. We used Hasty.ai (https://hasty.cloudfactory.com), a platform that offers annotation tools for computer vision tasks, to manually create bounding box annotations around any cages present in the imagery (see the Supplementary Materials for annotation guidelines). Each cage bounding box was also classified as denoting a circular cage, square cage, or other cage type, depending on the cage geometry. In total, our annotated data comprised 1775 circular cages, 689 square cages, and seven labels belonging to other cage types across 896 1024 × 1024 pixel images.

Once our images were labeled, we partitioned them into training, validation, and test sets for model training. As images belonging to the same mariculture facility are similar in appearance, we created the training splits by partitioning the mariculture locations in our training data to avoid data leakage. In this manner, we created the splits such that 20% of the mariculture locations were in the test set and 20% of the remaining locations were in the validation set, resulting in an overall train/validation/test split of 64/16/20% at the location level. In turn, this translated to an image-level split of 59/20/21% of the images used by the computer vision model during training. We also ensured that no images of French mariculture facilities entered into the training and validation sets to avoid data leakage for our downstream test application in the French Mediterranean (see the “Estimating marine finfish aquaculture tonnage in the French Mediterranean” section). A small fraction (1.4%) of the images in the training dataset belonged to ocean locations in France. However, we do not expect any data leakage from this subset, given that these images did not contain any mariculture facilities or any cages and given important differences between the GEP and the French aerial imagery (e.g., date of capture and image hue/saturation) that should prevent the model from gaining a performance advantage as a result of seeing these images during training.

By sampling images around known mariculture facility locations, we were able to quickly assemble a dataset containing a large number of mariculture cage instances. However, the resulting dataset only represents locations that are close to known mariculture production sites. A model trained on these data may perform well on this data distribution but poorly when asked to predict on a set of imagery that represents the complete distribution of coastal areas where mariculture activity is sought to be detected. For this reason, the 20% validation and 21% image-level test splits described above were used exclusively for model training and model selection rather than for the evaluation of model performance. Instead, to assess whether our model performs well in a realistic inference setting, we evaluated its capacity to detect mariculture cages from aerial imagery along the entirety of the French Mediterranean coast.

#### Model architecture and training

We used an object detection model to locate mariculture cages on the remote sensing imagery that we collected from GEP and on aerial imagery from IGN (*42*) (see the “Training data collection” and “Inference data collection” sections, respectively). Object detection models generate bounding boxes around instances of objects that they identify in an image. In contrast, previous studies using neural networks for aquaculture mapping have largely focused on image segmentation models [e.g., (*36*, *38*, *40*, *50*)], which classify each pixel of an image into a category. We opted for the object detection approach for its ease of use and adaptability, which is mainly illustrated in two parts of the training procedure. First, when labeling images with many small, geometrically complex features, such as mariculture cages, it can be faster to create bounding box labels than labels that perfectly resemble the object geometries. In other words, we expect that our model choice reduced the time cost of data collection. Second, there are a vast number of resources that enable practitioners to quickly and easily train well-performing object detection models, including platforms like YOLOv5 and off-the-shelf models that can be adapted to specific detection tasks.

In terms of model selection, we chose to fine-tune the medium-sized version of the YOLOv5 object detection architecture (yolov5m.pt) (*51*). Some models, such as YOLOv5, have been pretrained on large databases of images with bounding box labels [in our case, the MS COCO dataset; (*52*)]. Fine-tuning a pretrained model involves optimizing pretrained model weights to generate accurate predictions on additional data that represent a more specific prediction task. Fine-tuning models that have been trained on a diverse dataset of images and labels can lead to better model performance on a specific task, as opposed to training an entirely new model from scratch on the task without the use of pretrained weights, for two reasons. First, models trained to generate accurate predictions across a diverse set of images encode information that is relevant for many object detection tasks, which can lead to better performance (*53*). Second, by virtue of having encoded information that is useful across object detections tasks, pretrained models tend to require less data to achieve strong performance on a task than if the model was trained on randomly instantiated weights. YOLOv5 specifically has been widely used for a range of object detection tasks since its release in 2020 (*54*–*56*).

To train the model, we fine-tuned YOLOv5 for 50 epochs to generate predictions for three cage typologies (circular, square, and other) on 640 × 640 pixel images (YOLOv5 automatically resizes the 1024 × 1024 training images to this size), using a batch size of 16 and the default hyperparameters defined for YOLOv5. We use the validation and test sets comprising the GEP historical imagery to monitor the model’s performance and guide its training procedure, although we ultimately evaluate its performance on the French Mediterranean coastal imagery.

#### Prediction post-processing

We implemented two post-processing steps to improve the quality of our model predictions. First, we removed predictions that were located on land by constructing a shapefile of French landmass and filtering any predictions that intersected this geometry (see the “Constructing a French landmass shapefile” section in the Supplementary Materials). Second, we used the Density Based Spatial Clustering of Applications with Noise (DBSCAN) clustering algorithm (*57*) to aggregate our aquaculture cage predictions into cage clusters. DBSCAN represents each aquaculture cage as a node and defines edges between nodes that are within a user-specified distance of one another. The resulting connected components are the output clusters. We used the DBSCAN implementation from the scikit-learn package (version 0.0.post4) (*58*), which also allows users to filter clusters under a given size. As most aquaculture facilities operate a number of aquaculture cages in close proximity, filtering predictions that do not form a cluster of a given size is effective at removing false-positive predictions.

We tuned all hyperparameters related to output post-processing (the minimum cluster size and distance threshold used by the DBSCAN algorithm) and the selection of a model confidence threshold through the grid search procedure and fivefold cross-validation approach described in the Supplementary Materials, on 90% of the set of French aerial imagery containing ocean images (i.e., images that were not fully contained on French landmass). Using this procedure, we found the hyperparameters that maximized the product of recall and precision on the folds to be a model score threshold of 0.785, a distance threshold of 50 m, and a minimum cluster size of 5 cage predictions. Our final precision and recall measures assessing the prediction model’s performance were computed on an independent test set comprising the remaining 10% of the French aerial imagery containing ocean images.

### Estimating marine finfish aquaculture tonnage in the French Mediterranean

As our finfish cage prediction model was trained on a dataset curated from known mariculture locations, we evaluated its performance on a different dataset that is representative of the more realistic inference setting that this model could be deployed in to detect mariculture facilities and estimate production. For this inference dataset, we used aerial imagery along the French Mediterranean coast.

#### Inference data collection

We obtained imagery of the French Mediterranean coast for the 2000–2021 period from IGN, a French government agency that maintains geographical information. IGN provides high-resolution imagery for each French department (one of the country’s administrative subdivisions), captured with a cadence of 2 to 4 years on different years for varying regions within France (see the Supplementary Materials for a visualization of the imagery’s spatial coverage; fig. S4). The image resolution varies over time, ranging from 50 cm/pixel for most departments before 2014 to 15 cm/pixel in later years.

For our task, we collected imagery from IGN’s BD ORTHO series (*42*) along the French Mediterranean coast, covering nine departments (Pyrénées-Orientales, Aude, Hérault, Gard, Bouches-du-Rhône, Var, Alpes-Maritimes, Haute-Corse, and Corse-du-Sud). To do so, we created a shapefile of French Mediterranean coastal waters by intersecting a shapefile of the French Mediterranean sea with a shapefile of Europe’s shoreline buffered by 2000 m on each side (*59*, *60*). We then tiled our coastal waters shapefile into 200 m–by–200 m squares and queried the BD ORTHO data portal with each tile to download an image at the tile’s location. As the raw images from BD ORTHO are very large (6144 × 6144 pixels), we further tiled the downloaded images into 1024 × 1024 pixel squares before feeding them to the model.

Because of the different capture years on the imagery for each department, we were unable to obtain imagery for the complete French Mediterranean coast in a single year. For this reason, to generate annualized marine aquaculture tonnages, we combined the images from different years into groups (2000–2004, 2005–2009, 2010–2012, 2013–2015, 2016–2018, and 2019–2021) that comprise a spatial coverage of the coast that is as complete as possible (see fig. S4 for an example of the spatial distribution of imagery in the 2010–2012 period). Our tonnage estimation and uncertainty quantification procedure accounted for several artifacts stemming from the irregularity in the aerial imagery, including (i) the availability of multiple imagery from different years for a given location and (ii) missing imagery for a location within a given group.

#### Model performance evaluation

After running inference on the French Mediterranean coastal imagery, we evaluated our model’s performance by manually annotating a subset of the coastal imagery. To measure model precision, we annotated all of the images with model predictions. To allow for the estimation of model recall, we also annotated all images without predictions that were near known aquaculture production locations, as well as a random sample of images without predictions that were not near known sites. Table 1 describes how we partitioned the entire set of French aerial imagery into strata to define which images would be annotated. We first partitioned the images into two groups: those that had model predictions and those that did not. Next, for the images that did not have any predictions, we further disaggregated these into images that were within ~1 km of the aquaculture sites identified by Trujillo *et al.* (*29*) and those that were not. We note that, as distance calculations were performed using the coordinate reference system of the imagery, the threshold used to define whether a location was near a known site was, in practice, ~900 m rather than 1 km. In the table, we also present the images with predictions disaggregated according to the maximum model score of a prediction within the image.

**Table 1.** Stratification of the French aerial imagery. Stratification was based on the presence of model predictions and the location of the imagery [whether it was near any of the aquaculture locations found by Trujillo *et al.* (*29*)]. We exclude any aerial images that are fully contained within French landmass, such that all of the images across these strata have at least a partial view of the ocean.

Stratum	Number of images	Sampled images (%)	Number of cage predictions	Number of cages
Prediction: 0 ≤ maximum score < 0.3	2,203	100%	2,243	7
Prediction: 0.3 ≤ maximum score < 0.5	4,868	100%	5,316	16
Prediction: 0.5 ≤ maximum score < 0.8	3,402	100%	5,319	38
Prediction: 0.8 ≤ maximum score ≤ 1	1,157	100%	7,684	3,912
No prediction, near known location	6,846	100%	0	37
No prediction, not near known location	783,355	1%	0	0

To generate the sample for annotation, we sampled all of the images that contained model predictions. Then, for the images without predictions (the no-prediction strata), we sampled all of the images near known locations and 1% of the images that were not near known locations, as the latter stratum contained hundreds of thousands of images. Our prior was that the set of images without predictions and, far from known production locations, was highly unlikely to contain any aquaculture cages. However, by sampling a small fraction of the images in this stratum, we were able to estimate a conservative bound on the number of cage labels that may be in this group, despite random sampling not yielding any positive instances in these areas. To estimate this bound, we determined how large the proportion of images with cages could be in this stratum such that we could find, with a reasonable probability, zero cages in our sample (see the Supplementary Materials for further details). Our sampled images were labeled by CloudFactory (www.cloudfactory.com), a third-party data labeling vendor (see the Supplementary Materials for annotation guidelines). In total, human annotators reviewed 3.6% of the ~800,000 total images of the French Mediterranean coast during 2000–2021.

Our performance metrics of interest were model precision and recall. See the Supplementary Materials for additional information on how these metrics were computed and aggregated across strata.

#### Production estimation and uncertainty measurement

Our bounding box predictions of cages allowed us to estimate total finfish mariculture production over time with some uncertainty, following a similar methodology to that used in (*29*). Specifically, we model the annualized finfish tonnage (live weight equivalent), *Y_it_*, of a cluster of cages *i* detected in imagery from time period *t* using the following equation

$$Y_{\mathrm{it}}=A_{\mathrm{it}}\times D_{\mathrm{it}}\times S_{\mathrm{it}}\times H_{\mathrm{it}}\times \frac{1}{1000}$$ (1)

where *A_it_* is the estimated total cage area of the cluster (in square meters), *D_it_* is the estimated cage depth (in meters), *S_it_* is the estimated stocking density (in kilograms per cubic meter), *H_it_* is the estimated annual harvest frequency (e.g., *H_it_* = 2 for fish harvested every 6 months, reflecting that the same cage volume is re-used twice in a given year), and $\frac{1}{1000}$ is a conversion factor from kilograms to tonnes. Notably, our model assumes that cages are fully used within time periods relative to their stocking density and harvest frequency. Although we model uncertainty in these parameters, our tonnage computation does not account for periods of time in which cages may potentially be inactive.

To compute these estimates and their associated measures of uncertainty, we developed a framework that allowed us to make distributional assumptions on each of the four production factors and propagate the uncertainty from these to compute a point estimate and SD for the tonnage in each period. This framework enabled us to model the uncertainty in each production factor independently and to incorporate uncertainty from the following sources to generate more robust tonnage estimates for each period: (i) the computer vision model’s performance; (ii) artifacts of the French Mediterranean coastal imagery, resulting in multiple aerial images for a given location; and (iii) cage area uncertainty due to a lack of knowledge of the underlying cage orientation within each detected bounding box (see the “Cage area calculations from bounding boxes” section in the Supplementary Materials). Our approach used the method of composition (*61*), which generates independent and identically distributed samples of an output variable across multiple iterations. In each iteration, samples are first drawn from the distributions of a set of input variables and are then used to compute the output variable. In our application, for each cluster of cages, we independently sampled values for each of the production factors from the distributions defined in the Supplementary Materials (see the “Production factor distributions for tonnage uncertainty quantification” section) and combined these to compute the cluster’s annualized tonnage for the period using Eq. 1.

Then, we computed period-level annualized tonnage estimates for the entire region by summing the cluster-level tonnage estimates in each time period. We performed 10,000 iterations of this procedure, such that our final period-level estimates and error bars reflect the mean annualized tonnage and its SD, respectively, across the samples from these iterations. We note that, as we grouped the imagery from each year into periods representing a near complete survey of the French Mediterranean coast (2000–2004, 2005–2009, 2010–2012, 2013–2015, 2016–2018, and 2019–2021), these measures are estimates of the region’s annualized aquaculture production during each period. See the Supplementary Materials for further details and a visualization (fig. S3) of the production factor distributions used to generate the samples for the input variables in each iteration.

Although we grouped the French aerial imagery such that we could obtain coverage of the coast that was as complete as possible, in some cases, there was missing imagery that barred us from obtaining a representation of the entire coast. In particular, the aerial imagery was unavailable for some locations in the 2000–2004 (imagery area covers 82% of the imagery area from the most complete period), 2013–2015 (78%), and 2016–2018 (90%) time periods. For this reason, we performed an imputation exercise in which we estimated the annualized production that could potentially be missing from the locations that lacked imagery in a time period. This was accomplished by measuring the tonnage output from these locations using the imagery from a different time period with more complete imagery. Specifically, we used the 2005–2009 period to impute the tonnage from locations with missing imagery for the 2000–2004 period and used the 2010–2012 period, which had relatively good spatial coverage, to impute missing tonnage for both the 2013–2015 and the 2016–2018 periods. By using the information from other time periods, our estimates capture whether the missing imagery in an imputed time period belongs to locations that are amenable to aquaculture and are, thus, relatively more likely to have production compared to locations that did not experience any production during the comparison period. We emphasize that, rather than point estimates at the year level, the measures of production presented in Fig. 6 reflect annualized production over the wave of imagery in each time period and, in the case of the missing imagery estimates, estimates of average production over a wider period of time that includes the comparison period used to impute the estimates.

In addition to the prediction-based estimates of production described thus far, we showcased the use of our method in a HITL setting that illustrates how our model could be used to perform more efficient yet still expert-driven surveys of aquaculture production in a region. In this case, tonnage estimates were computed using the cage annotations derived from the stratified sample of images from Table 1 (3.6% of the entire set of imagery), instead of using the predicted cage polygons obtained from our model over the entire set of imagery. The procedure to compute the tonnage estimates and uncertainty measures is exactly the same as the one outlined in this section for the model estimates, with the exception that, in the HITL case, we do not incorporate uncertainty from model performance, as the polygons were human verified. We also used the human cage annotations to estimate an upper bound on the total number of cages in the coastal imagery from 2000 to 2021, which we discuss in the “Estimating an upper bound on the population of aquaculture cages” section of the Supplementary Materials.

We compared the prediction-based and HITL-based annualized production estimates to annual FAO aquaculture production data for France in the Mediterranean and Black Sea region, within marine environments, for all finfish species (meagre, seabream, seabass, and miscellaneous marine fishes). FAO point estimates were computed as the average annual FAO values within each period, while error bars in Fig. 6 reflect the SD of the annual production statistics that fall in each time period.

## Supplementary Materials

### This PDF file includes:

Supplementary Text Figs. S1 to S7 Tables S1 and S2 References
