# Supplementary material for: Remote sensing and computer vision for marine aquaculture
Source: Sci Adv. 2024 Oct 16;10(42):eadn4944. doi: 10.1126/sciadv.adn4944 (PMC11482319; doi:10.1126/sciadv.adn4944)
Supplement: Supplementary file 1 — Supplementary Text Figs. S1 to S7 Tables S1 and S2 References [file sciadv.adn4944_sm.pdf]

Science Advances
AAAS

Supplementary Materials for

Remote sensing and computer vision for marine aquaculture

Sebastian Quaade *et al.*

Corresponding author: Kit T. Rodolfa, krodolfa@stanford.edu; Daniel E. Ho, deho@stanford.edu

Sci. Adv. **10**, eadn4944 (2024)
DOI: 10.1126/sciadv.adn4944

This PDF file includes:

Supplementary Text
Figs. S1 to S7
Tables S1 and S2
References

Supplementary Text

Cage annotation protocol from imagery

In this section, we describe our procedure to annotate finfish cages from aerial and satellite imagery using bounding boxes, in order to create the datasets used to train the object detection model and to evaluate model performance in the French Mediterranean. While we generated the dataset for model training by following this protocol ourselves, to generate the French Mediterranean dataset we employed CloudFactory – an IT services company that offers HITL solutions for machine learning and business purposes – as our third-party annotator. Given a remote sensing image as input, the task consisted of two steps:

1. Annotators first had to identify whether any marine finfish cages were present in the image and, if any cages were present, to determine the correct cage type (square or circular). To aid annotators in this identification process, we developed a flow chart (Fig. S5) that begins by determining whether a body of water is present in the image and whether physical structures can be observed within this water body, and outlines the physical characteristics of these structures that can be used to determine whether they are in fact square or circular finfish cages. In cases where it was challenging to determine whether an object was a marine finfish cage, annotators were asked to use GEP to inspect the location, querying for imagery that was as close as possible to the date of the provided image.

2. Annotators had to draw individual bounding boxes around each identified marine finfish cage. In order to compute precise area estimates and uncertainty measures, these bounding boxes were required to be as tight as possible around each square and circular cage.

We provided annotators with several examples of images for different steps along the flow chart. Additionally, we offered guidance on the annotation procedure for images showing more difficult annotation cases, such as the presence of non-finfish rectangular cages, the presence of cages on land, and the presence of cages in lower-quality imagery. Fig. S6 visualizes a subset of these examples.

Constructing a French landmass shapefile

We filter false positive model predictions on land by constructing a shapefile representing French landmass and removing any predictions that intersect with its geometry. To do so, we perform a series of geometric manipulations using a coarse 10km x 10km resolution outline of French territory (including naval territory) (*62*) and a high resolution shapefile of the European coastline from the European Environment Agency (*60*), as well as a coarse shapefile of French marine territory from Flanders Marine Institute (*59*). First, we take the difference of the polygon representing the entire French territory and the polygon representing French marine areas to obtain a coarse outline of French landmass. Next, we dissect the coarse French landmass geometry with the high resolution outline of the European coast, yielding disjoint polygons whose

boundaries are high resolution representations of the locations where French land meets French ocean. We inspect these polygons manually, and combine those that correspond to land into a single multipolygon.

Evaluating model performance

We characterized the performance of our model by measuring model precision and estimating model recall using the stratified sample of annotated images. Let I be the population of images on which labels Y and predictions $\hat{Y}$ reside. We partitioned the set of images into the $n = 6$ strata defined in Table 1, which we denote as $I_1, \ldots, I_6$. We annotated all of the images in $I_1, ..., I_5$, and a random sample S_6 of the images from I_6. For any set of images J, let Y_J and $\hat{Y}_J$ be the labels and predictions on the images, respectively.

To measure precision and estimate recall, our quantities of interest were the empirical ratio of true positive predictions to the total number of predictions and to the total number of labels, respectively. Let u be the bounding box of a model prediction (label) and let V be the set of bounding boxes belonging to a set of labels (predictions). We defined a true positive instance as any time the bounding box of a label and prediction overlapped, as described in the following equation:

$$TP(u; V) := \begin{cases} 1 & \text{if} \quad \sum_{v \in V} \mathbb{1}[u \cap v] > 0 \\ 0 & \text{otherwise} \end{cases} \tag{S1}$$

We measure precision and recall both at the cage and cage cluster-level. In the case of cages, we defined whether an instance was a true positive using the detected or annotated bounding boxes for the cage. In the case of cage clusters, we defined whether an instance was a true positive using the bounding box given by the union of all cage-level detected or annotated bounding boxes belonging to a cluster. For both cages and cage clusters, we emphasize that we only considered overlap between predictions and labels belonging to the same image – and thus, belonging to the imagery from the same *year* – to define a true positive instance.

To measure cage-level and cage cluster-level precision we simply computed the ratio of true positive predictions to total predictions, given that we annotated all of the images that had model predictions:

$$PR = \frac{1}{|\hat{Y}|} \sum_{\hat{y} \in \hat{Y}} TP(\hat{y}; Y) \tag{S2}$$

We computed cage-level and cage cluster-level recall as follows. As we annotated all of the

im-

ages belonging to $I_1, ..., I_5$, we estimated population-level recall by measuring recall in $I_1, ..., I_5$ and estimating recall in I_6 using the sample of images S_6. We used the number of labels in each stratum as weights to aggregate these estimates to the population level, as follows:

$$\begin{aligned}\widehat{RE} &= \frac{|Y_{I_{1-5}}|}{|Y|} RE_{1-5} + \frac{|Y_{S_6}|}{|Y|} \widehat{RE}_6 \\ &= \frac{|Y_{I_{1-5}}|}{|Y|} \sum_{y \in Y_{I_{1-5}}} \frac{1}{|Y_{I_{1-5}}|} TP(y; \hat{Y}) + \frac{|Y_{S_6}|}{|Y|} \sum_{y \in Y_{S_6}} \frac{1}{|Y_{S_6}|} TP(y; \hat{Y})\end{aligned} \tag{S3}$$

Note that since $|Y_{S_6}| = 0$ (see Table 1), it follows that $|Y_{I_{1-5}}| = |Y|$ and so our estimate of recall is further simplified to the following equation:

$$\widehat{RE} = \frac{1}{|Y|} \sum_{y \in Y} TP(y; \hat{Y}) \tag{S4}$$

Estimating an upper bound on the population of aquaculture cages

Our stratum including images that have no predictions and are more than one kilometer away from a location belonging to the dataset of mariculture sites of (*29*) – the *No prediction, not near known location* stratum in Table 1 – contained more images than we were able to manually inspect for aquaculture cages. We randomly sampled 1% of these images for manual inspection and found no aquaculture cages on them. However, the small size of the sample relative to the stratum population meant there was a non negligible likelihood that we drew a random sample with no aquaculture cages, despite their presence in the population. As a result, the population of aquaculture cages may have actually been larger than what we found from fully sampling the other strata.

We estimate an upper bound on the number of cages in this stratum as follows. First, we estimate how large the proportion of images with cages could be in this stratum such that we could find, with a 50% probability, zero cages in a sample of images of size $m = 10,518$ – the number of images that we actually sampled from this stratum. By drawing $10,000$ binomial samples of size m for each proportion $p \in \{0.000010, 0.000011, ..., 0.00009, 0.0001\}$, we find that the proportion of images that have cages could be no larger that $p = 0.00007$ in order to have a sample with zero labels, with a 50% probability. With this proportion, we estimate an upper bound on the number of cages in the *No prediction, not near known location* stratum as $p \times |I_6| \times k$ cages, where $k = 5$ is the average number of cages per image.

Tuning of the detection post-processing procedure

We tuned the model score threshold, as well as the maximum distance and minimum cluster size parameter for the DBSCAN algorithm using a broad grid search. We used a k-fold cross-

validation approach in which we partitioned 90% of the images in our data that were classified as not belonging to land into five folds. Per this approach, we used each fold as a tuning dataset and the remaining folds as a training dataset. We post-processed the predictions in the tuning set using each possible combination of the following parameter values, and then estimated precision and recall with the resulting predictions on the true labels in the tuning set:

$$CT = \{0.600, 0.605, 0.610, ..., 0.995, 1.000\} \quad \text{(S5)}$$

$$DT = \{10, 30, 50, , ..., 130, 150\} \quad \text{(S6)}$$

$$MC = \{1, 2, 3, 4, 5, 6, 7, 8, 9, 10\} \quad \text{(S7)}$$

where CT is the set of explored confidence thresholds, DT is the set of explored distance thresholds (in meters), and MC is the set of explored minimum cluster sizes. To select the optimal combination of post-processing parameters, we chose the combination that maximized the product of precision and recall of the post-processed predictions. For robustness, we also explored using the combination that maximized the F1 score, and found that both metrics retrieved the same hyperparameter choices.

Finally, to characterize their true performance, we computed the precision and recall of model predictions in a test dataset comprising the remaining 10% of the data, after post-processing the predictions using the selected hyperparameters.

Cage area calculations from bounding boxes

In this section, we outline the computation of the area estimate, minimum area and maximum area of each cage from a bounding box of width w and height h (in meters), according to the cage type (square or circular). Given that the model's detected bounding boxes and the annotated bounding boxes need not be perfectly square, we compute the underlying cage area for circular cage predictions using area calculations for ellipses, and compute the underlying cage area for square cage predictions using area calculations for rectangles. The area calculations for each cage type are summarized in Table S1 and visualized in Fig. S7.

For circular cages that are not on the border of an image, we assume that the height and width of the bounding box are equal to the length of the ellipse's major and minor axes. The surface area of a circular cage is then given by:

$$A_{circular} = \pi(w/2)(h/2) = \pi wh/4 \quad \text{(S8)}$$

As the bounding box width and height perfectly identify the ellipse area, these estimates have

zero uncertainty, and so the maximum and minimum cage area are equal to the central estimate.

However, bounding boxes of circular cages that are not fully contained in an image do not necessarily identify the ellipses' principal axes. In these instances, we assume that true cage surface area is uniformly distributed between the minimum and maximum surface area of a partial ellipse that is bounded by the bounding box. When a bounding box is in the corner of an image, the minimum possible surface area occurs when the bounding captures an ellipse chord that approximates a diagonal from one corner of the bounding box to the other; the maximum possible surface area occurs when the bounding box captures a perfect quarter ellipse. When a bounding box is located on one edge of an image, the minimum possible surface area occurs when the ellipse approximates a triangle circumscribed in the bounding box, and the maximum possible surface area occurs when the ellipse approximates the rectangular shape of the bounding box. In both of these cases, the bounds of the distribution of a cage's true surface area are given by:

$$A_{circular}^{max} = \frac{\pi w h}{4} \tag{S9}$$

$$A_{circular}^{min} = \frac{wh}{2} \tag{S10}$$

We then derive the central estimate using the properties of a uniform distribution:

$$A_{circular} = \frac{(A_{max} + A_{min})}{2} \tag{S11}$$

We also treat the orientation of a square cage within a bounding box as a random draw from a uniform distribution. In this case, the minimum possible surface area occurs when the square cage's vertices touch the midpoint of the bounding box's edges. On the other hand, the maximum area occurs when the square cage is equivalent to the bounding box.

$$A_{square}^{max} = wh \tag{S12}$$

$$A_{square}^{min} = \frac{wh}{2} \tag{S13}$$

Since we assume that the rotation angle of the underlying cage within a bounding box is uniform, the central estimate is then given by:

$$A_{square} = \frac{3}{4}wh = \frac{1}{2} \times \frac{wh}{2} + \frac{1}{2} \times wh \tag{S14}$$

Production factor distributions for tonnage uncertainty quantification

For a cluster of cages *i* detected in imagery from time period *t*, we defined the distribution of each production factor used to draw samples for the cluster's tonnage Y_{it}. This cluster-level tonnage was then used to generate a sample for Y_t, the finfish tonnage in each period, by summing over the cluster-level tonnage for all detected clusters in a time period. Fig. S3 visualizes example distributions of each of the four production factors (cage area, cage depth, stocking density and harvest frequency), which were defined as follows:

Cage area

Cage area samples were drawn from the following Uniform distribution:

$$A_{it} \sim U(A_{it}^{min}, A_{it}^{max}) \tag{S15}$$

where A_{it}^{min} is the minimum total cage area of cluster *i* from time period *t* and A_{it}^{max} is the maximum. The stochasticity for the cage area combined three sources of uncertainty: *(1)* model uncertainty regarding the cage predictions; *(2)* uncertainty regarding the true cage area, given that we capture rectangular bounding boxes around the cages using our detection model; and *(3)* uncertainty in the image selection for locations that have multiple imagery from different years due to overlap in the spatial coverage of the annual IGN imagery. Each of these sources contributed to defining the bounds of the Uniform distribution from Equation S15. Let j be a cage prediction from cage cluster i from time period t of type $c \in \{square, circular\}$ that is output by our prediction model. Then, let A_{cjit} be the estimated area of the underlying cage, computed from the width and height of the output bounding box as outlined in Table S1.

First, to incorporate model prediction error, we began by estimating the distributions of the model errors using our cage predictions and the annotations from the stratified sample. We stratified our predictions according to the cage type c and period t. Then, for each cage j predicted by our model, we found the annotated cage k of type c from period t with the highest spatial overlap, and computed the error as the difference between the area of the annotated cage and that of the predicted cage. For each cage type and time period, we used these errors to fit a Normal distribution, $N(\mu_{ct}, \sigma_{ct})$. We fit 12 Normal distributions in total, reflecting the 2 cage types and 6 time periods. Once we computed these error distributions, we adjusted the estimated area of each cage j of type c from period t as follows:

$$\tilde{A}_{cjit} = A_{cjit} + \epsilon_{cjit}, \text{ where } \epsilon_{cjit} \sim N(\mu_{ct}, \sigma_{ct}) \tag{S16}$$

We ensured that $\tilde{A}_{cjit} > 0$ by sampling the errors until this condition was met for all cages.

Second, to capture uncertainty in the actual surface cage area, we leveraged our classification of cage predictions as circular or square to obtain estimates of the minimum and maximum possible underlying cage area for each prediction. From the adjusted cage area $\tilde{A}_{cjit}$, we obtained the upper and lower bounds reflecting the maximum and minimum cage area, $\tilde{A}^{min}_{cjit}$ and $\tilde{A}^{max}_{cjit}$, depending on the cage type c, using the computation outlined in Table S1. From this step, we obtained an aggregate upper and lower bound on the cage area of cage cluster *i* from time period *t* as follows:

$$\tilde{A}^{min}_{it} = \sum_{j \in \text{ cluster } i} \tilde{A}^{min}_{cjit} \tag{S17}$$

$$\tilde{A}^{max}_{it} = \sum_{j \in \text{ cluster } i} \tilde{A}^{max}_{cjit} \tag{S18}$$

Third, to capture uncertainty in the image selection within time periods, we began by optimizing the selection of the imagery. To do so, we selected the combination of all available images from different years that resulted in the maximum and minimum cage area for each location along the coast. This image selection defines which specific cage predictions are incorporated into the tonnage estimates. Let $s \in \{imagemax, imagemin\}$ denote this image selection scheme. For each image selection scheme, we separately computed the upper and lower bounds on the aggregate cage area for the cluster, $\tilde{A}^{min}_{cjit}(s)$ and $\tilde{A}^{max}_{cjit}(s)$. It is important to note that to ensure that the adjusted cage area under selection scheme $s = imagemin$ was less or equal to that of $s = imagemax$, we sampled errors at the cage-level, such that the same cage suffers the same model error adjustment under both schemes.

Finally, we combine the minimum and maximum aggregate cage areas under each image selection scheme to compute the upper and lower bounds on the Uniform distribution from Equation S15 from which we will sample the cage area for cluster i:

$$A^{min}_{it} = \tilde{A}^{min}_{it}(s = imagemin) \tag{S19}$$

$$A^{max}_{it} = \tilde{A}^{max}_{it}(s = imagemax) \tag{S20}$$

Cage depth

Cage depth samples were drawn from a mixture of two truncated Normal distributions:

$$D_{it} \sim \kappa TN(\mu_l = \hat{d}_i, \sigma_l = \sigma_{li}, a_l = 1, b_l = \hat{d}_i) + (1-\kappa)TN(\mu_r = \hat{d}_i, \sigma_r = \sigma_{ri}, a_r = \hat{d}_i, b_r = 2\hat{d}_i) \tag{S21}$$

where $\kappa \in [0, 1]$ defines the mixture component weights, μ_w defines the Truncated Normal mean, σ_w defines the Truncated normal standard deviation, and a_w and b_w define the lower and upper bounds of the Truncated Normal. We define these for $w \in \{l, r\}$, the Truncated Normal components on the left-hand and right-hand side of $\hat{d}_i$, our estimate of the cage-depth for a cluster of cages i.

We modeled this distribution according to the bathymetry data for each cluster of cages and according to FAO cage aquaculture guidelines. These guidelines recommend that the cage depth should be no larger than half of the water depth (*63*). While the guidelines are not mandatory, facilities have strong incentives to maximize the cage depth within a close neighborhood of this threshold due to the risk of cage abrasion when water levels are low. Cages are tethered to the ocean floor such that they rise and lower with the change in water levels, which implies that cage damage could occur at sufficiently low water levels if the cage depth is too high. The mixture of Truncated Normal distributions models this optimization problem faced by facilities: cage depth should ideally be as close to the threshold as possible to maximize production, and cage depth can indeed go above the recommended threshold but not too far above due to potential risks of abrasion. The use of a mixture in the distribution for cage depth allowed us to better model this asymmetry in the probability mass of the cage depth.

To define the distribution in Equation S21 for cluster i, we first computed an estimate of the water depth at the location of the cluster, z_i, using 2022 bathymetry data from the European Marine Observation and Data Network (*64*). As the resolution of the bathymetry data is somewhat coarse (115m x 115m), and some cage clusters may be very close to shore, we estimated z_i as the maximum bathymetry value observed within the cage detections belonging to a cluster. Where bathymetry data was unavailable (this was the case for 21 out of 136 cage clusters), we used an average cage depth estimate of 4.84m, which was derived from (*29*) as the average cage depth for France. Additionally, we imposed a minimum threshold for the cage depth of 1 meter ($\bar{d} = 1$), to be conservative in our uncertainty measures. We used z_i to estimate the cage cluster-level cage depth recommended by FAO, $\hat{d}_i$ as follows:

$$\hat{d}_i = \begin{cases} \max\{\frac{z_i}{2}, \bar{d}\} \text{ if } z_i \text{ is available at cluster } i \\ 4.84 \text{ if } z_i \text{ is unavailable at cluster } i \end{cases} \tag{S22}$$

Then, the mixture of Truncated Normal distributions from Equation S21 defines a cage depth

distribution over the interval between a_l, the minimum cage depth (assumed to be 1 meter), and $b_r = 2 \times \hat{d_i}$, the estimated water depth at the cluster. For both truncated Normal distributions, we chose the standard deviation for a cluster to be $\sigma_{li} = \sigma_{ri} = \frac{b-a}{1.96}$. This models the spread of the Truncated Normal distribution to be proportional to that of a Normal distribution Z such that $P(Z < a_l) = 0.025$ in the case of the left-hand Truncated Normal, and such that $P(Z > b_r) = 0.025$ in the case of the right-hand Truncated Normal. We chose $\kappa = 0.5$.

Stocking density
Stocking density samples were drawn from the following truncated Normal distribution:

$$S_{it} \sim TN(\mu = \mu_t, \sigma = \sigma_t, a = 5, b = 20) \tag{S23}$$

where μ and σ are the mean and standard deviation of the Truncated Normal, and a and b are its lower and upper bounds, respectively. The upper bound of the truncated Normal was set to $a = 20 kg/m^3$, according to the technical rules that oversee mariculture activities in each French department. This is the maximum average stocking density established in the technical rules of most departments that have finfish aquaculture production activities (*e.g.*, Var (*65*) and Bouches-du-Rhône (*66*)). On the other hand, the lower bound of the truncated Normal, b, was based on literature describing commercial aquaculture practices (*67*).

For the mean and standard deviation of this distribution, we inferred parameter values from the grey literature for three finfish species: seabass, seabream and meagre. These species represent most of France's finfish marine aquaculture production, accounting for 83.2% of the country's marine finfish production in the Mediterranean in 2021 (*26*). The remaining 16.8% of marine finfish aquaculture production in 2021 was classified in FAO statistics under "marine fishes nei" or miscellaneous marine fishes; to compute the parameter values using the production shares, we re-classified the tonnage from this category as seabream. The mean and standard deviations for each species derived from the literature are summarized in Table S2.

We aggregated these species-level parameters into period-level stocking density and harvest frequency parameter values using the annual share of aquaculture production across species as weights. Species-level production statistics for each time period were derived from FishStatJ, FAO's platform for fishery and aquaculture statistics (*26*). Let $X_{s,t}$ be the marine production of species s in time period t (in tonnes), and let X_t be the total marine finish production in time period t. Then, we estimated the mean and variance of production factor ρ_t at time period t from the species-level factors $\rho_{s,t}$ as follows:

$$\bar{\rho}_t = \sum_{s \in S} \frac{X_{s,t}}{X_t} \rho_{s,t} \tag{S24}$$

$$Var(\bar{\rho}_t) = \sum_{s \in S} (\frac{X_{s,t}}{X_t})^2 \times Var(\rho_{s,t}) \tag{S25}$$

Harvest frequency
Annual harvest frequency samples were drawn from the following Normal distribution:

$$H_{it} \sim N(\mu = \mu_t, \sigma = \sigma_t) \tag{S26}$$

As in the case of the stocking density, μ_t and σ_t were computed as the weighted average of the harvest frequency and harvest frequency standard deviation, respectively, of each species according to the production weights of each species in a given time period.

Fig. S1

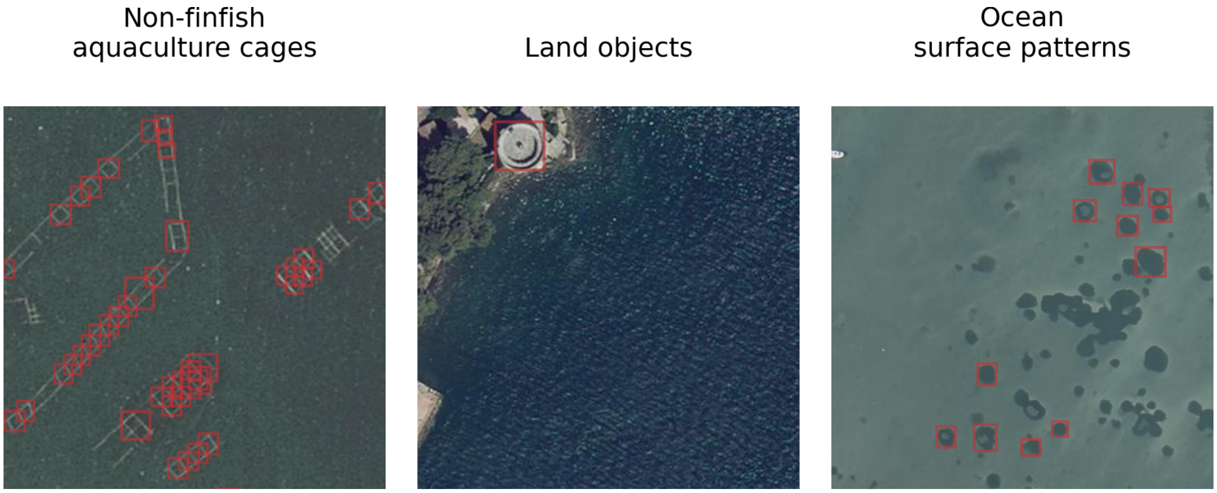

Fig. S1: **Examples of common false positive predictions from the object detection model.** Common false positive predictions (red bounding boxes) from the detection model in the French Mediterranean aerial imagery include non-finfish aquaculture cages (*e.g.*, shellfish rafts or other aquatic raft structures), circular structures on water and land, and other water-based objects such as boats and boat yards. The two post-processing steps (land filtering and cage clustering) that complement our detection model improve overall precision through the removal of some of these false positive instances. Imagery: IGN (*42*)

Fig. S2

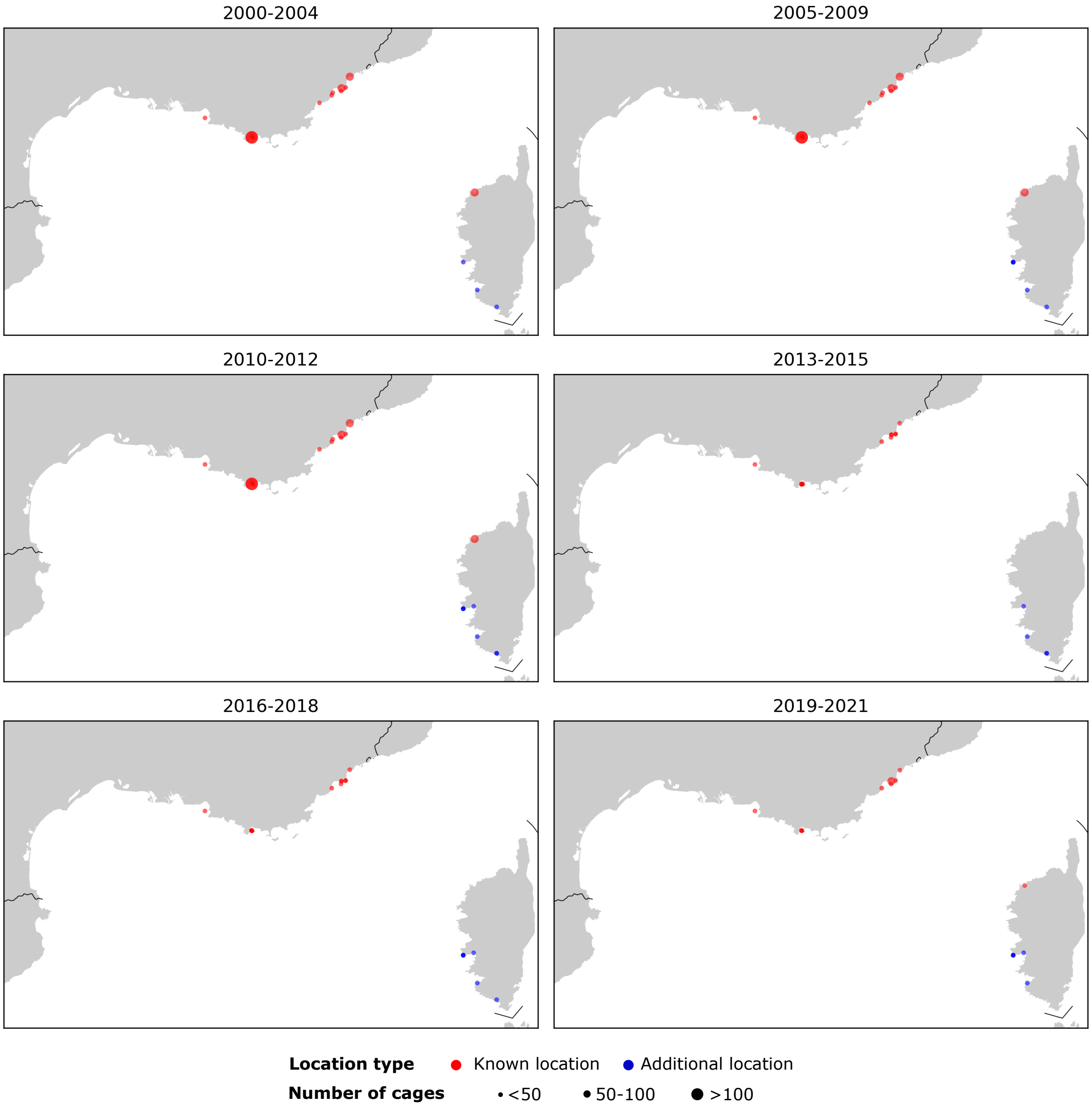

Fig. S2: **Marine finfish aquaculture production locations in the French Mediterranean over time.** Red points indicate the known locations found by (*29*) in their manual survey of Google Earth during 2002-2010. Blue points indicate cage clusters detected by our model that are at least one kilometer away from these known locations.

Fig. S3

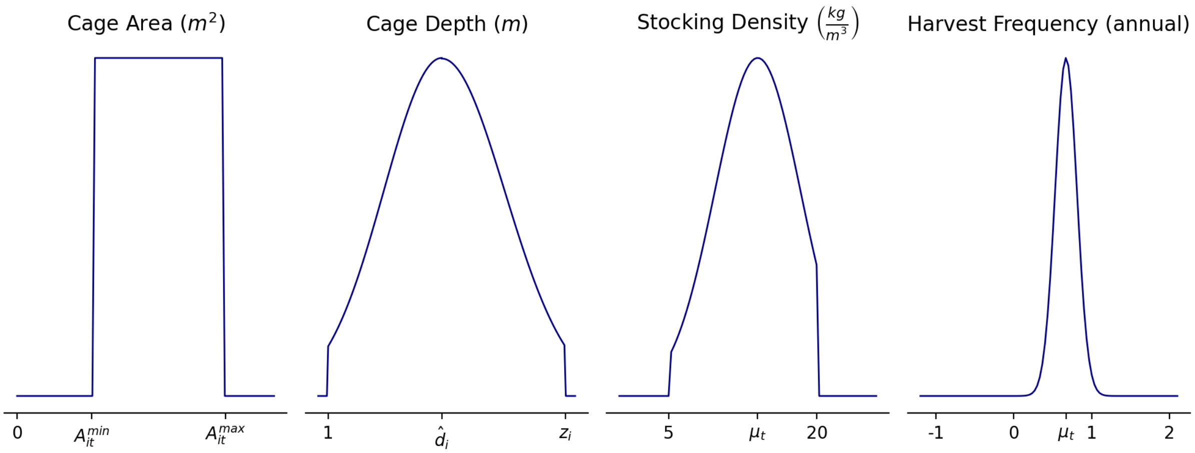

Fig. S3: **Example distributions of the production factors used in tonnage estimation.** The distributions for each of the four production factors used to compute finfish aquaculture tonnage estimates and uncertainty measures were designed according to bathymetry constraints, FAO guidelines for cage culture, French regulation on mariculture practices, and finfish life cycles.

Fig. S4

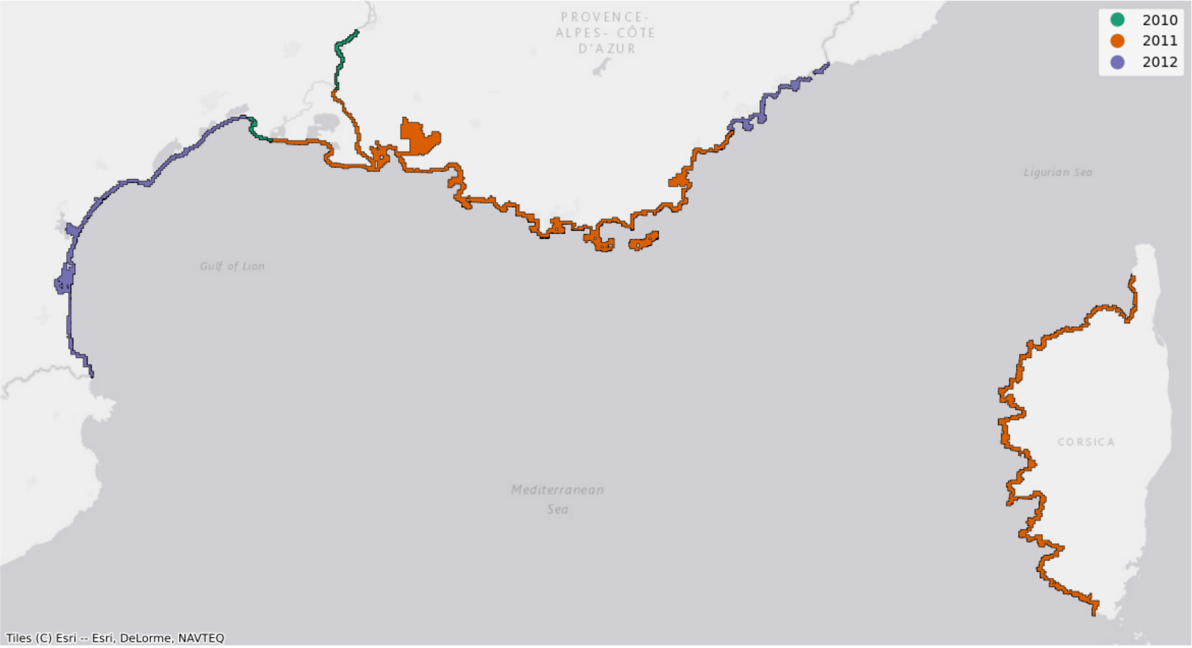

Fig. S4: **Spatial coverage of the aerial imagery of the French Mediterranean.** The imagery – provided by IGN (*42*) – at each location is captured at a cadence of approximately 2-4 years, and is captured in different years for different regions along the coast. The figure shows the spatial coverage belonging to 2010, 2011 and 2012, whose imagery we combine to generate annualized finfish mariculture production estimates for the 2010-2012 period for the entire French Mediterranean.

Fig. S5

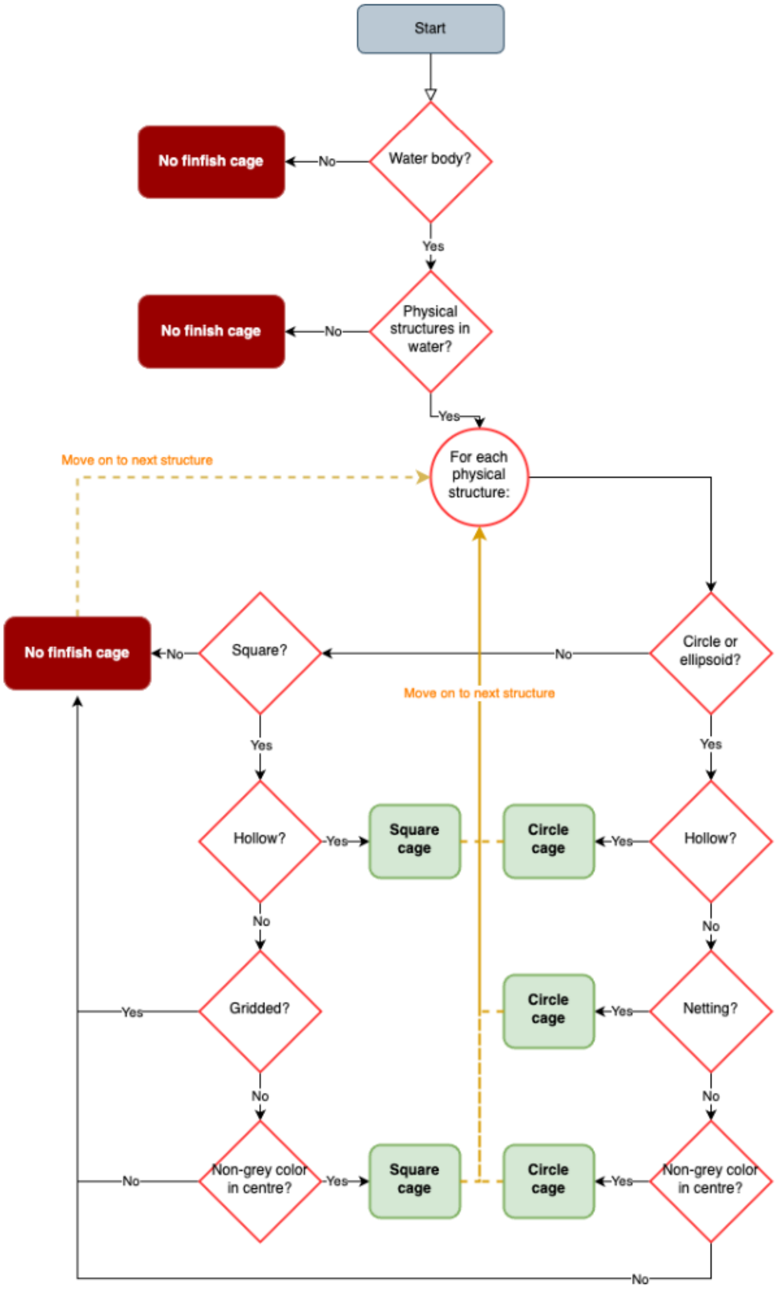

Fig. S5: **Annotation protocol to identify marine finfish cages.** Given a remote sensing image, annotators used this protocol to determine whether there were any marine finfish cages in the image, and what type of cage structure (square or circular) these represented.

Fig. S6

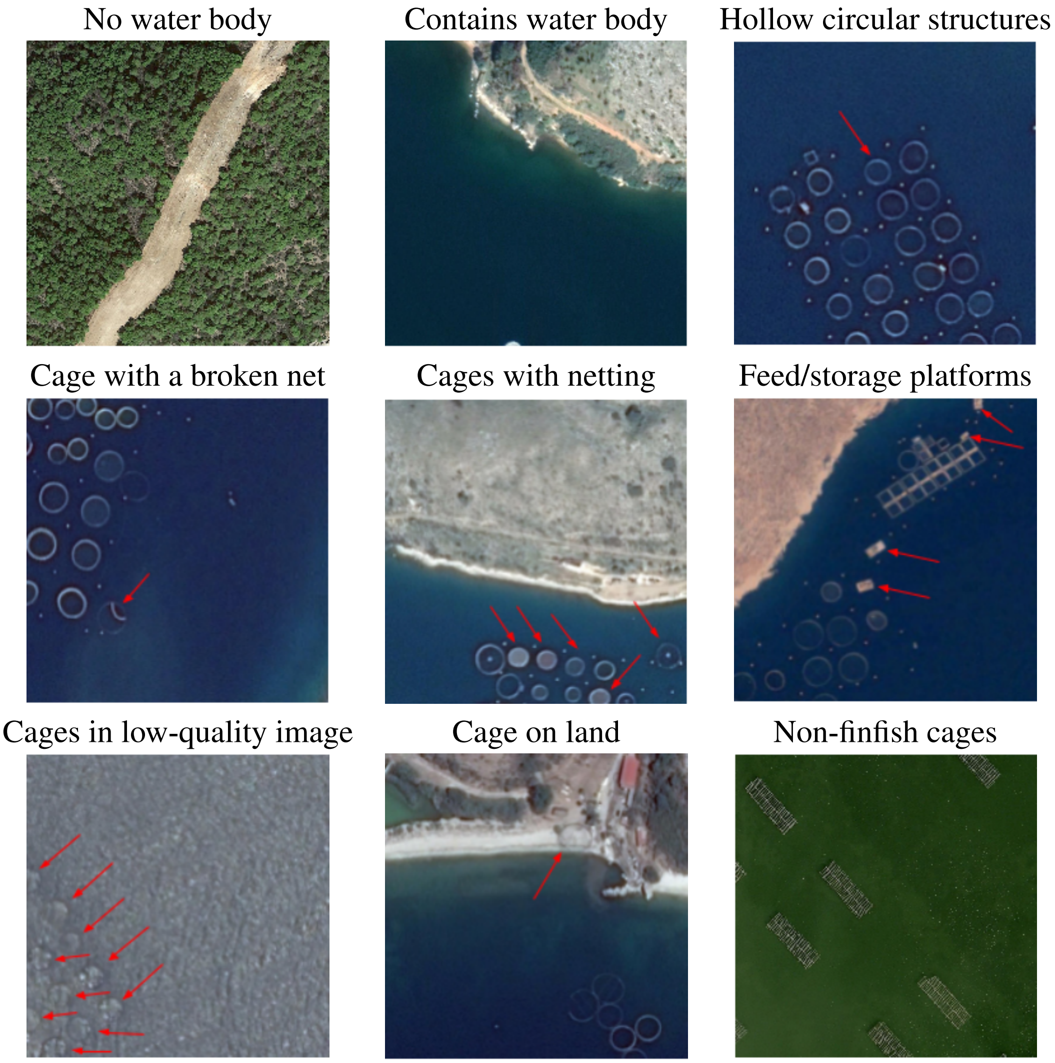

Fig. S6: **Examples provided in the annotation protocol**. Annotators were provided examples of different images containing land, water bodies, and finfish cages, as well as difficult edge cases such as images containing non-finfish, rectangular cages. Imagery: Google, 2023 CNES/Airbus (*41*)

Fig. S7

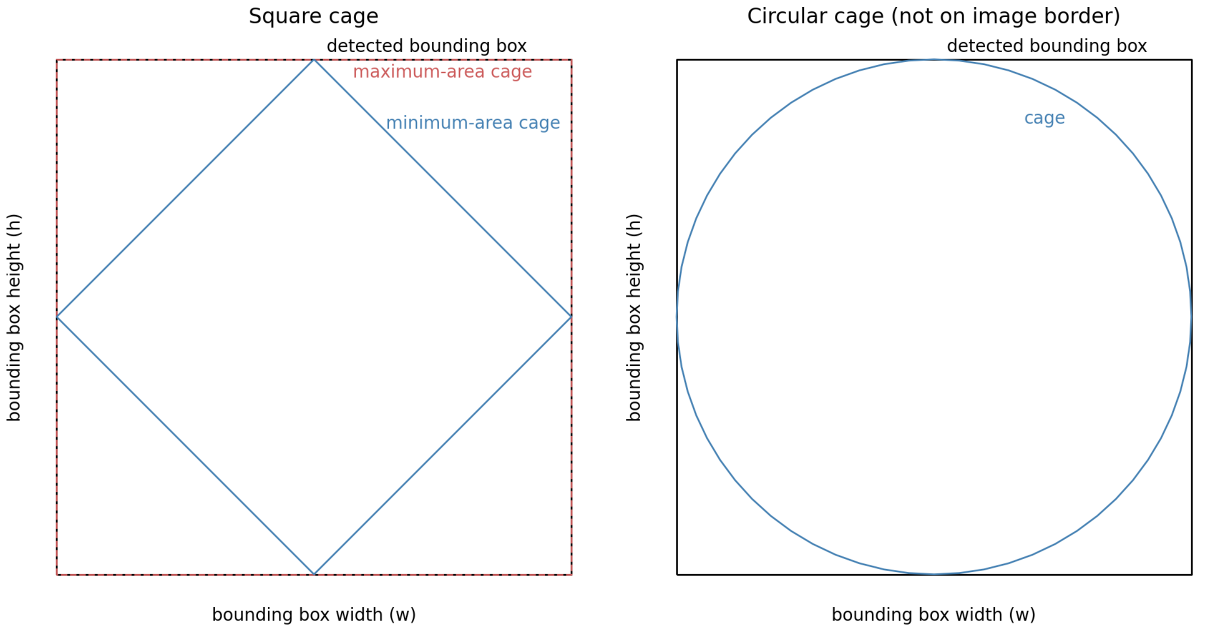

Fig. S7: **Calculation of the underlying cage area from rectangular bounding boxes.** Our cage area estimates from square or circular cage detections reflect uncertainty in the underlying cage rotation within a bounding box.

Table S1

**Cage type** (c)	**Maximum cage area** (A_c^{max})	**Minimum cage area** (A_c^{min})	**Area estimate** (A_c)
**Circular**			
Not on the image border	$\frac{\pi wh}{4}$	$\frac{\pi wh}{4}$	$\frac{\pi wh}{4}$
On the image border	$\frac{\pi wh}{4}$	$\frac{wh}{2}$	$\frac{1}{2} \times (A_c^{max} + A_c^{min})$
**Square**	wh	$\frac{wh}{2}$	$\frac{3wh}{4}$

Table S1: **Cage area computation from model-predicted or human-annotated bounding boxes.** Cage area estimate, minimum cage area and maximum cage area estimates from the height (h) and width (w) of each bounding box (in meters) according to the cage type and location of the bounding box within the image.

Table S2

**Factor** and *Species*	**Trujillo et al. (*29*) estimate**	**Estimate**	**Range**	**Standard deviation**	**Source**
**Stocking density** (kg/m^3)					
Meagre	NA	15.00	10-15	1.44	(*68*)
Sea bass	12.00	20.00	5-20	4.33	(*67, 69*)
Sea bream	12.00	12.50	5-20	4.33	(*67, 70*)
**Harvest frequency** *(annual)*					
Meagre	NA	0.67	0.5-1.0	0.14	(*70, 71*)
Sea bass	0.67	0.60	0.5-1.2	0.20	(*72–74*)
Sea bream	0.75	0.67	0.5-1.2	0.20	(*72–74*)

Table S2: **Estimates and uncertainty measures for species-level stocking density and harvest frequency.** We include (*29*)'s estimates for comparison. Standard deviations were derived for the estimate assuming a uniform distribution over the parameter range found in the literature.

REFERENCES AND NOTES

1. FAO, *The State of World Fisheries and Aquaculture 2022: Towards Blue Transformation* (FAO, 2022).

2. D. Klinger, R. Naylor, Searching for solutions in aquaculture: Charting a sustainable course. *Annu. Rev. Env. Resour.* **37**, 247–276 (2012).

3. M. Troell, R. L. Naylor, M. Metian, M. Beveridge, P. H. Tyedmers, C. Folke, K. J. Arrow, S. Barrett, A.-S. Crépin, P. R. Ehrlich, A. Gren, N. Kautsky, S. A. Levin, K. Nyborg, H. Österblom, S. Polasky, M. Scheffer, B. H. Walker, T. Xepapadeas, A. de Zeeuw, Does aquaculture add resilience to the global food system? *Proc. Natl. Acad. Sci. U.S.A.* **111**, 13257–13263 (2014).

4. R. R. Gentry, H. E. Froehlich, D. Grimm, P. Kareiva, M. Parke, M. Rust, S. D. Gaines, B. S. Halpern, Mapping the global potential for marine aquaculture. *Nat. Ecol. Evol.* **1**, 1317–1324 (2017).

5. C. Costello, L. Cao, S. Gelcich, M. Cisneros-Mata, C. M. Free, H. E. Froehlich, C. D. Golden, G. Ishimura, J. Maier, I. Macadam-Somer, T. Mangin, M. C. Melnychuk, M. Miyahara, C. L. De Moor, R. Naylor, L. Nøstbakken, E. Ojea, E. O'Reilly, A. M. Parma, A. J. Plantinga, S. H. Thilsted, J. Lubchenco, The future of food from the sea. *Nature* **588**, 95–100 (2020).

6. C. D. Golden, J. Z. Koehn, A. Shepon, S. Passarelli, C. M. Free, D. F. Viana, H. Matthey, J. G. Eurich, J. A. Gephart, E. Fluet-Chouinard, E. A. Nyboer, A. J. Lynch, M. Kjellevold, S. Bromage, P. Charlebois, M. Barange, S. Vannuccini, L. Cao, K. M. Kleisner, E. B. Rimm, G. Danaei, C. DeSisto, H. Kelahan, K. J. Fiorella, D. C. Little, E. H. Allison, J. Fanzo, S. H. Thilsted, Aquatic foods to nourish nations. *Nature* **598**, 315–320 (2021).

7. J. A. Gephart, P. J. G. Henriksson, R. W. R. Parker, A. Shepon, K. D. Gorospe, K. Bergman, G. Eshel, C. D. Golden, B. S. Halpern, S. Hornborg, M. Jonell, M. Metian, K. Mifflin, R. Newton, P. Tyedmers, W. Zhang, F. Ziegler, M. Troell, Environmental performance of blue foods. *Nature* **597**, 360–365 (2021).

8. C. E. Boyd, A. A. McNevin, R. P. Davis, The contribution of fisheries and aquaculture to the global protein supply. *Food Sec.* **14**, 805–827 (2022).

9. A. B. Dauda, A. Ajadi, A. S. Tola-Fabunmi, A. O. Akinwole, Waste production in aquaculture: Sources, components and managements in different culture systems. *Aquac. Fish.* **4**, 81–88 (2019).

10. D. P. Bureau, K. Hua, Towards effective nutritional management of waste outputs in aquaculture, with particular reference to salmonid aquaculture operations. *Aquacult. Res.* **41**, 777–792 (2010).

11. L. Ren, J. Zhang, J. Fang, Q. Tang, M. Zhang, M. Du, Impact of shellfish biodeposits and rotten seaweed on the sediments of Ailian Bay, China. *Aquac. Int.* **22**, 811–819 (2014).

12. J. Wang, A. H. W. Beusen, X. Liu, A. F. Bouwman, Aquaculture production is a large, spatially concentrated source of nutrients in chinese freshwater and coastal seas. *Environ. Sci. Technol.* **54**, 1464–1474 (2020).

13. D. Schar, E. Y. Klein, R. Laxminarayan, M. Gilbert, T. P. Van Boeckel, Global trends in antimicrobial use in aquaculture. *Sci. Rep.* **10**, 21878 (2020).

14. R. Lulijwa, E. J. Rupia, A. C. Alfaro, Antibiotic use in aquaculture, policies and regulation, health and environmental risks: A review of the top 15 major producers. *Rev. Aquac.* **12**, 640–663 (2020).

15. P. J. Ashley, Fish welfare: Current issues in aquaculture. *Appl. Anim. Behav. Sci.* **104**, 199–235 (2007).

16. F. S. Conte, Stress and the welfare of cultured fish. *Appl. Anim. Behav. Sci.* **86**, 205–223 (2004).

17. C. Brown, C. Dorey, Pain and emotion in fishes–fish welfare implications for fisheries and aquaculture. *Anim. Studies J.* **8**, 175–201 (2019).

18. B. Franks, C. Ewell, J. Jacquet, Animal welfare risks of global aquaculture. *Sci. Adv.* **7**, eabg0677 (2021).

19. FAO, Global aquaculture production (Fisheries and Aquaculture Division, 2023); www.fao.org/fishery/en/collection/aquaculture.

20. National Marine Fisheries Service, Fisheries of the United States, 2019 (U.S. Department of Commerce, NOAA, 2021); https://media.fisheries.noaa.gov/2021-05/FUS2019-FINAL-webready-2.3.pdf.

21. B. Campbell, D. Pauly, Mariculture: A global analysis of production trends since 1950. *Mar. Policy* **39**, 94–100 (2013).

22. C. Espinosa-Miranda, B. Cáceres, O. Blank, M. Fuentes-Riquelme, S. Heinrich, Entanglements and mortality of endemic chilean dolphins (Cephalorhynchus eutropia) in salmon farms in Southern Chile. *Aquat. Mamm.* **46**, 337–343 (2020).

23. H. Heredia-Azuaje, E. J. Niklitschek, M. Sepúlveda, Pinnipeds and salmon farming: Threats, conflicts and challenges to co-existence after 50 years of industrial growth and expansion. *Rev. Aquac.* **14**, 528–546 (2022).

24. A. E. Harnish, R. W. Baird, E. Corsi, A. M. Gorgone, D. Perrine, A. Franco, C. Hankins, E. Sepeta, Long-term associations of common bottlenose dolphins with a fish farm in Hawaii and impacts on other protected species. *Mar. Mamm. Sci.* **39**, 794–810 (2023).

25. M. Tigchelaar, J. Leape, F. Micheli, E. H. Allison, X. Basurto, A. Bennett, S. R. Bush, L. Cao, W. W. L. Cheung, B. Crona, F. DeClerck, J. Fanzo, S. Gelcich, J. A. Gephart, C. D. Golden, B. S. Halpern, C. C. Hicks, M. Jonell, A. Kishore, J. Z. Koehn, D. C. Little, R. L. Naylor, M. J. Phillips, E. R. Selig, R. E. Short, U. R. Sumaila, S. H. Thilsted, M. Troell, C. C. C. Wabnitz, The vital roles of blue foods in the global food system. *Glob. Food Sec.* **33**, 100637 (2022).

26. FAO, FAO Fisheries and Aquaculture - FishStatJ - Software for fishery statistical time series (FAO Fisheries and Aquaculture Division, 2020); www.fao.org/fishery/en/knowledgebase/150.

27. European Marine Observation and Data Network (EMODnet), EMODnet human activities, aquaculture, marine finfish (EMODnet Human Activities, 2021); https://ows.emodnet-humanactivities.eu/geonetwork/srv/api/records/03c35b79-808f-4168-9d30-2de44a55a6f4.

28. G. Clawson, C. D. Kuempel, M. Frazier, G. Blasco, R. S. Cottrell, H. E. Froehlich, M. Metian, K. L. Nash, J. Többen, J. Verstaen, D. R. Williams, B. S. Halpern, Mapping the spatial distribution of global mariculture production. *Aquaculture* **553**, 738066 (2022).

29. P. Trujillo, C. Piroddi, J. Jacquet, Fish farms at sea: The ground truth from google earth. *PLOS ONE* **7**, e30546 (2012).

30. G. Katselis, K. Tsolakos, J. A. Theodorou, Mapping of greek marine finfish farms and their potential impact on the marine environment. *J. Mar. Sci. Eng.* **10**, 286 (2022).

31. M. Ottinger, K. Clauss, C. Kuenzer, Opportunities and challenges for the estimation of aquaculture production based on earth observation data. *Remote Sens.* **10**, 1076 (2018).

32. T. Zhang, X. Yang, S. Hu, F. Su, Extraction of coastline in aquaculture coast from multispectral remote sensing images: Object-based region growing integrating edge detection. *Remote Sens.* **5**, 4470–4487 (2013).

33. Y. Liu, X. Yang, Z. Wang, C. Lu, Z. Li, F. Yang, Aquaculture area extraction and vulnerability assessment in Sanduao based on richer convolutional features network model. *J. Oceanol. Limnol.* **37**, 1941–1954 (2019).

34. C. Handan-Nader, D. E. Ho, Deep learning to map concentrated animal feeding operations. *Nat. Sustain.* **2**, 298–306 (2019).

35. C. Robinson, B. Chugg, B. Anderson, J. M. L. Ferres, D. E. Ho, Mapping industrial poultry operations at scale with deep learning and aerial imagery. *IEEE J. Sel. Top. Appl. Earth Obs. Remote Sens.* **15**, 7458–7471 (2022).

36. T. Shi, Q. Xu, Z. Zou, Z. Shi, Automatic raft labeling for remote sensing images via dual-scale homogeneous convolutional neural network. *Remote Sens.* **10**, 1130 (2018).

37. Y. Fu, J. Deng, H. Wang, A. Comber, W. Yang, W. Wu, S. You, Y. Lin, K. Wang, A new satellite-derived dataset for marine aquaculture areas in China's coastal region. *Earth Syst. Sci. Data* **13**, 1829–1842 (2021).

38. Z. Zou, C. Chen, Z. Liu, Z. Zhang, J. Liang, H. Chen, L. Wang, Extraction of aquaculture ponds along coastal region using U2-net deep learning model from remote sensing images. *Remote Sens.* **14**, 4001 (2022).

39. H. Su, S. Wei, J. Qiu, W. Wu, RaftNet: A new deep neural network for coastal raft aquaculture extraction from landsat 8 OLI data. *Remote Sens.* **14**, 4587 (2022).

40. Y. Fu, Z. Ye, J. Deng, X. Zheng, Y. Huang, W. Yang, Y. Wang, K. Wang, Finer resolution mapping of marine aquaculture areas using worldView-2 imagery and a hierarchical cascade convolutional neural network. *Remote Sens. (Basel)* **11**, 1678 (2019).

41. Google, Google Earth Pro, versions 7.3.4.8573–7.3.6.9326, Google Earth (2023); https://earth.google.com.

42. Institut national de l'information géographique et forestière, BD ORTHO, version 2.0, République Française Géoservices (2023); https://geoservices.ign.fr/documentation/donnees/ortho/bdortho.

43. GLOBEFISH (Food and Agriculture Organization of the United Nations), European Seabass and Gilthead seabream - March 2009 (Food and Agriculture Organization of the United Nations, 2009); www.fao.org/in-action/globefish/market-reports/resource-detail/en/c/338075/.

44. M. Sievers, Ø. Korsøen, F. Warren-Myers, F. Oppedal, G. Macaulay, O. Folkedal, T. Dempster, Submerged cage aquaculture of marine fish: A review of the biological challenges and opportunities. *Rev. Aquac.* **14**, 106–119 (2022).

45. Z. Zeng, D. Wang, W. Tan, G. Yu, J. You, B. Lv, Z. Wu, RCSANet: A full convolutional network for extracting inland aquaculture ponds from high-spatial-resolution images. *Remote Sens.* **13**, 92 (2021).

46. Z. Zeng, D. Wang, W. Tan, J. Huang, Extracting aquaculture ponds from natural water surfaces around inland lakes on medium resolution multispectral images. *Int. J. Appl. Earth Obs. Geoinf.* **80**, 13–25 (2019).

47. Y. Han, J. Huang, F. Ling, J. Qiu, Z. Liu, X. Li, C. Chang, H. Chi, Dynamic mapping of inland freshwater aquaculture areas in jianghan plain, China. *IEEE J. Sel. Top. Appl. Earth Obs. Remote Sens.* **16**, 4349–4361 (2023).

48. M. Burke, A. Driscoll, D. B. Lobell, S. Ermon, Using satellite imagery to understand and promote sustainable development. *Science* **371**, eabe8628 (2021).

49. Guest Contributor, Petition to save Poros Island from aquacultural industrialization, *Greek City Times*, 6 December 2020; https://greekcitytimes.com/2020/12/06/petition-to-save-poros.

50. Y. Fu, S. You, S. Zhang, K. Cao, J. Zhang, P. Wang, X. Bi, F. Gao, F. Li, Marine aquaculture mapping using GF-1 WFV satellite images and full resolution cascade convolutional neural network. *Int. J. Digit. Earth* **15**, 2048–2061 (2022).

51. G. Jocher, YOLOv5 by Ultralytics, GitHub (2020); https://github.com/ultralytics/yolov5.

52. T.-Y. Lin, M. Maire, S. Belongie, J. Hays, P. Perona, D. Ramanan, P. Dollár, C. L. Zitnick, Microsoft COCO: Common objects in context, paper presented at the 13th European Conference on Computer Vision, Zurich, Switzerland, 6 to 12 September 2014.

53. X. Han, Z. Zhang, N. Ding, Y. Gu, X. Liu, Y. Huo, J. Qiu, Y. Yao, A. Zhang, L. Zhang, W. Han, M. Huang, Q. Jin, Y. Lan, Y. Liu, Z. Liu, Z. Lu, X. Qiu, R. Song, J. Tang, J.-R. Wen, J. Yuan, W. X. Zhao, J. Zhu, Pre-trained models: Past, present and future. *AI Open* **2**, 225–250 (2021).

54. W. Wu, H. Liu, L. Li, Y. Long, X. Wang, Z. Wang, J. Li, Y. Chang, Application of local fully Convolutional Neural Network combined with YOLO v5 algorithm in small target detection of remote sensing image. *PLOS ONE* **16**, e0259283 (2021).

55. Y. Fang, X. Guo, K. Chen, Z. Zhou, Q. Ye, Accurate and automated detection of surface knots on sawn timbers using YOLO-V5 model. *Bioresources* **16**, 5390–5406 (2021).

56. M. Kasper-Eulaers, N. Hahn, S. Berger, T. Sebulonsen, Ø. Myrland, P. E. Kummervold, Detecting heavy goods vehicles in rest areas in winter conditions using YOLOv5. *Algorithms* **14**, 114 (2021).

57. M. Ester, H.-P. Kriegel, J. Sander, X. Xu, A density-based algorithm for discovering clusters in large spatial databases with noise, paper presented at the Second International Conference on Knowledge Discovery and Data Mining, Portland, OR, 2 to 4 August 1996.

58. F. Pedregosa, G. Varoquaux, A. Gramfort, V. Michel, B. Thirion, O. Grisel, M. Blondel, P. Prettenhofer, R. Weiss, V. Dubourg, J. Vanderplas, A. Passos, D. Cournapeau, M. Brucher, M. Perrot, E. Duchesnay, Scikit-learn: Machine learning in Python. *J. Mach. Learn. Res.* **12**, 2825–2830 (2011).

59. Flanders Marine Institute, Maritime Boundaries and Exclusive Economic Zones (200NM), version 11, Maritime Boundaries Geodatabase (2019); https://doi.org/10.14284/386.

60. European Environment Agency, Europe Coastline Shapefile, Environmental Information Systems (2015); www.eea.europa.eu/ds%5Fresolveuid/06227e40310045408ac8be0d469e1189.

61. M. A. Tanner, *Tools for Statistical Inference: Methods for the Exploration of Posterior Distributions and Likelihood Functions*, Springer Series in Statistics (Springer, 1996).

62. European Environment Agency, France Shapefile, Environmental Information Systems (2013); www.eea.europa.eu/ds%5Fresolveuid/ca88b5c9fb874aa3a3d53e2a84c3e12d.

63. F. Cardia, A. Ciattaglia, R. A. Corner, *Guidelines and Criteria on Technical and Environmental Aspects of Cage Aquaculture Site Selection in the Kingdom of Saudi Arabia* (Food and Africulture Organization of the United Nations, 2017); www.fao.org/3/i6719e/i6719e.pdf.

64. European Marine Observation and Data Network (EMODnet), EMODnet digital bathymetry (DTM 2022), EMODnet Product Catalogue (2022);

https://emodnet.ec.europa.eu/geonetwork/srv/eng/catalog.search#/metadata/b5278b56-00f0-4fcf-9955-76d4b4880bdb.

65. Direction départementale des territoires et de la mer du Var, “Arrêté Préfectoral du 30 Oct 2020 portant schéma des structures des exploitations de cultures marines du département du Var” (Préfet Du Var, 2020); www.var.gouv.fr/contenu/telechargement/26928/184361/file/ss%5F2020%5Fsigne%5Fprefet.pdf.

66. Direction départementale des territoires et de la mer et des Bouches-du-Rhône, “Arrêté portant schéma des structures des exploitations de cultures marines du département des Bouches-du-Rhône” (Préfet des Bouches-du-Rhône, 2023); www.bouches-du-rhone.gouv.fr/content/download/15879/99974/file/Projet%5FSdS%5FBdR.pdf.

67. Scientific Opinion of the Panel on Animal Health and Welfare on a request from the European Commission, Animal welfare aspects of husbandry systems for farmed european seabass and gilthead seabream. *EFSA J.* **844**, 1–21 (2023).

68. M. Monfort, Present market situation and prospects of meagre (Argyrosomus regius), as an emerging species in Mediterranean aquaculture (General Fisheries Commission for the Mediterranean, 2010); www.fao.org/3/i1675e/i1675e.pdf.

69. Improving the welfare of European sea bass and gilthead sea bream (Compassion in Food Business, n.d.); www.compassioninfoodbusiness.com/media/7436996/the-science-driving-change-for-gilthead-sea-bream-and-european-sea-bass.pdf.

70. N. L. François, M. Jobling, C. Carter, P. Blier, Eds., *Finfish Aquaculture Diversification* (CABI, 2010).

71. N. Kružić, B. Mustać, I. Župan, S. Čolak, Meagre (*Argyrosomus regius* Asso, 1801) aquaculture in Croatia. *Croat. J. Fish.* **74**, 14–19 (2016).

72. B. García García, C. Rosique Jiménez, F. Aguado-Giménez, J. García García, Life cycle assessment of seabass (*Dicentrarchus labrax*) produced in offshore fish farms: Variability and multiple regression analysis. *Sustainability* **11**, 3523 (2019).

73. M. Zoli, L. Rossi, C. Bibbiani, J. Bacenetti, Life cycle assessment of seabass and seabream production in the Mediterranean area: A critical review. *Aquaculture* **573**, 739580 (2023).

74. K. Abdou, F. Ben Rais Lasram, M. S. Romdhane, F. Le Loc'h, J. Aubin, Rearing performances and environmental assessment of sea cage farming in Tunisia using life cycle assessment (LCA) combined with PCA and HCPC. *Int. J. Life Cycle Assess.* **23**, 1049–1062 (2018).
